# Novel Double Factor PGT strategy analyzing blastocyst stage embryos in a single NGS procedure

**DOI:** 10.1371/journal.pone.0205692

**Published:** 2018-10-17

**Authors:** Javier del Rey, Francisco Vidal, Lorena Ramírez, Nina Borràs, Irene Corrales, Iris Garcia, Olga Martinez-Pasarell, Silvia F. Fernandez, Raquel Garcia-Cruz, Aïda Pujol, Alberto Plaja, Itziar Salaverria, Maria Oliver-Bonet, Jordi Benet, Joaquima Navarro

**Affiliations:** 1 Unitat de Biologia Cel·lular i Genètica Mèdica, Facultat de Medicina, Departament de Biologia Cel·lular, Fisiologia i Immunologia, Universitat Autònoma de Barcelona, Bellaterra, Spain; 2 Congenital Coagulopathies, Blood and Tissue Bank, Barcelona, Spain; 3 Molecular Diagnosis and Therapy, Vall d’Hebron Research Institute, Universitat Autònoma de Barcelona (VHIR-UAB), Barcelona, Spain; 4 CIBER de Enfermedades Cardiovasculares (CIBERCV), ISCIII, Madrid, Spain; 5 Fundació Puigvert, Hospital de Sant Pau i de la Santa Creu, Barcelona, Spain; 6 Center for Embryo Medicine, Barcelona, Spain; 7 Centro de Infertilidad y Reproducción Humana (CIRH)- Eugin group, Barcelona, Spain; 8 Àrea de Genètica Clínica i Molecular, Hospital Vall d'Hebron, Barcelona, Spain; 9 Hematopathology Unit, Hospital Clinic, Institut d'Investigacions Biomèdiques August Pi Sunyer, Centro de Investigación Biomédica en Red de Cáncer, Barcelona, Spain; Peking University Third Hospital, CHINA

## Abstract

In families at risk from monogenic diseases affected offspring, it is fundamental the development of a suitable Double Factor Preimplantation Genetic Testing (DF-PGT) method for both single-gene analysis and chromosome complement screening. Aneuploidy is not only a major issue in advanced-maternal-age patients and balanced translocation carriers, but also the aneuploidy rate is extremely high in patients undergoing in vitro fertilization (IVF), even in young donors. To adequate NGS technology to the DF-PGT strategy four different whole genome amplification systems (Sureplex, MALBAC, and two multiple displacement amplification systems-MDA) were tested using TruSight One panel on cell lines and blastocyst trophectoderm biopsies-TE. Embryo cytogenetic status was analyzed by Nexus software. Sureplex and MALBAC DNA products were considered not suitable for PGT diagnosis due to inconsistent and poor results on Trusight one (TSO) panel. Results obtained with both MDA based methods (GEH-MDA and RG-MDA) were appropriate for direct mutation detection by TSO NGS platform. Nevertheless, RG-MDA amplification products showed better coverage and lower ADO rates than GEH-MDA. The present work also demonstrates that the same TSO sequencing data is suitable not only for the direct mutation detection, but also for the indirect mutation detection by linkage analysis of informative SNPs. The present work also demonstrates that Nexus software is competent for the detection of CNV by using with TSO sequencing data from RG-MDA products, allowing for the whole cytogenetic characterization of the embryos. In conclusion, successfully development of an innovative and promising DF-PGT strategy using TSO-NGS technology in TE biopsies, performed in-house in a single laboratory experience, has been done in the present work. Additional studies should be performed before it could be used as a diagnostic alternative in order to validate this approach for the detection of chromosomal aneuploidies.

## Introduction

Preimplantation genetic diagnosis (PGT) has been applied in more than 200 monogenic disorders.[1] Aneuploidy is the leading cause for embryo arrest, implantation failure, recurrent spontaneous abortions and congenital defects.[2–4] Moreover, a high incidence of aneuploidy has also been reported in embryos from *in vitro* fertilization cycles[3,5,6] and in oocytes, even from young donors.[4,7,8] The Double Factor-PGT (DF-PGT) allows to transfer not only healthy embryos, free from the monogenic disease, but also embryos with the highest implantation potential according to its cytogenetic characterization.[9,10] This should produce an increase of pregnancy and birth rates in PGT cycles and consequently should reduce the incidence in the populations of genetic illnesses. An increasing number of studies have focused on developing new technologies for embryo screening in order to select the most viable for transfer.[11] These comprehensive cytogenetic techniques, such as metaphase comparative genomic hybridization (mCGH), single-nucleotide polymorphism arrays (SNP-arrays) or array-CGH (aCGH), have allowed the detection of both aneuploidies and segmental chromosome imbalances in oocytes,[12] cleavage-stage embryos[12–14] and blastocysts.[15,16]

Recently, in embryo analysis, Next-Generation Sequencing (NGS) has been proven to be useful in the identification of family mutations and *de novo* mutation in embryo biopsies.[17,18] Moreover, current advances in NGS are providing new methods for the comprehensive cytogenetic screening of the embryos. [19]

All these comprehensive analysis techniques applied to PGT require DNA amplification and, for this reason, several whole genome amplification (WGA) techniques have been used.[20–24] When working with limited amount of DNA, the success relays on the WGA protocol used. The chosen WGA DNA product must be representative of the cell genome. Bias introduced during this amplification process may lead to misinterpretations. To minimize the allele drop-out (ADO) rate and the amplification artifacts, Trophectoderm (TE) biopsies are being widely favored over the single blastomere analysis. Moreover, this reduces the errors caused by mosaicism.

The main goal of this study is to adapt TruSight One Sequencing (TSO) panel, which analyzes 4,813 disease-associated genes, to the DF-PGT from TE biopsies. This approach should make feasible not only the direct detection of family mutations, but also the indirect through linkage analysis of heterozygous Single Nucleotide Polymorphisms (SNPs) as well as the evaluation of the cytogenetic status of the embryo based on TSO sequencing data in a single workflow.

## Material and methods

### Sample recruitment and preparation

Fibroblasts cell line GM03184, 47, XY, +15, (Coriell) and lymphocyte cell line GM07381, 46, XY (Coriell) were used as positive aneuploidy control and as reference DNA for mCGH analysis, respectively. Pools containing 8 cells were isolated with the use of a 170-μm denuding pipette (Cook Ireland). Genomic DNA was obtained using the QuickExtract Kit (Epicentre) according to the manufacturer’s protocol.

**Fig 1:**
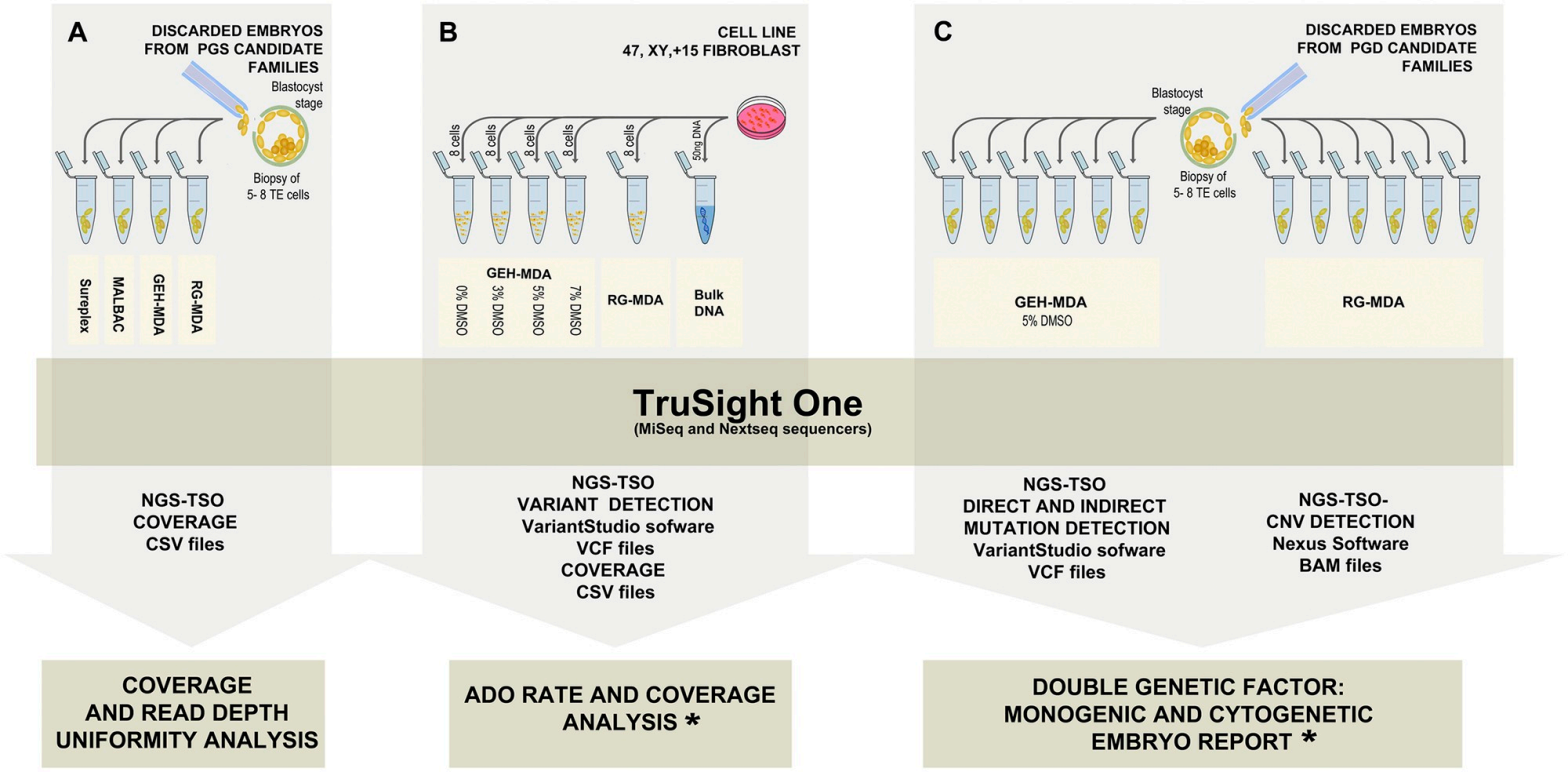
Design of the study for DF-PGT with NGS-TSO. A) Evaluation of TSO sequencing panel outcome using the product of four different WGA systems (Sureplex, MALBAC, GEH-MDA and RG-MDA) in F11E1-S, F12E1-M, F7E1-GEH and F13E1-RG embryos. B) Evaluation of ADO and coverage with MDA-based methods in GM03184 cell line. C) Translational application of the NGS-TSO panel to DF-PGT, in embryos F1E1-GEH, F2E1-GEH, F3E1-GEH, F4E1-GEH, F5E1-GEH, F6E1-GEH, F1E2-RG, F2E2-RG, F3E2-RG, F8E1-RG, F9E1-RG and F10E1-RG. * The validation of NGS-CNV results was performed by CGH approaches.

Extracted DNA from the GM03184 cell line was used to determine the ADO and coverage results obtained from NGS-TSO using the products of the different WGA methods tested (Fig 1).

Sixteen discarded embryos from 13 families undergoing PGT were included in this study. Four embryos were used for the optimization of the WGA protocol in NGS-TSO platform (Fig 1). Three of them were aneuploid embryos discarded after Preimplantation Genetic Testing for aneuploidies (PGT-A) (F7E1-GEH, F11E1-S and F12E1-M). The fourth one (F13E1-RG) was a discarded embryo after a Preimplantation Genetic Testing for Monogenic disorders (PGT-M) due to be undiagnosed. For the validation of the DF-PGT approach by using TSO, twelve embryos were used. Eleven of them presented a mutation associated to a monogenic disease and the remaining one was an undiagnosed embryo from a previous PGT-M cycle (Table 1 and Fig 1).

**Table 1:** Disease and mutation details of the embryos included in DF-PGT strategy with TSO panel.

Embryo	Affected by	Disorder/Locus MIM number	Gene	HGVSc	HGVSp	Inh
F1E1-GEH	Basal cell nevus syndrome	109400/601309	*PTCH1*	c.2776delT	p.Trp926GlyfsTer36	AD
F2E1-GEH	Exostoses Multiple type I	133700/608177	*EXT1*	c.1468delC	p.Leu490TrpfsTer9	AD
F3E1-GEH	Hereditary Breast and Ovarian Cancer	114480/113705	*BRCA1*	c.68_69delAG	p.Glu23ValfsTer17	AD
F4E1-GEH	Machado-Joseph disease	109150/607047	*ATXN3*	c.892_894CAG[70]	p.Gln298_Gln305[23]	AD
F5E1-GEH	Pyruvate kinase deficiency of red cells	266200/609712	*PKLR*	c.721G>T	p.Glu241Ter	AR
				c.204delC	p.Phe69SerfsTer39	
F6E1-GEH	Exudative vitreoretinopathy 1	133780/604579	*FZD4*	c.1501_1502delCT	p.L501SfsX33	AD
F1E2-RG	Basal cell nevus syndrome	109400/601309	*PTCH1*	c.2776delT	p.Trp926GlyfsTer36	AD
F2E2-RG	Exostoses Multiple type I	133700/608177	*EXT1*	c.1468delC	p.Leu490TrpfsTer9	AD
F3E2-RG	Hereditary Breast and Ovarian Cancer	114480/113705	*BRCA1*	c.68_69delAG	p.Glu23ValfsTer17	AD
F8E1-RG	Colorectal cancer, hereditary nonpolyposis, type 2	609310/120436	*MLH1*	c.199G>A	p.Gly67Arg	AD
F9E1-RG	Familial Adenomatous Polyposis 1	175100/611731	*APC*	c.4611_4612delAG	p.Glu1538IlefsTer5	AD
F10E1-RG	Familial Adenomatous Polyposis 1	175100/611731	*APC*	c.1660C>T	p.Arg554Ter	AD

F(n), Family; E(n), Embryo; GEH, GE Healthcare MDA; RG, REPLI-g MDA; AD, Autosomal dominant; AR, autosomal recessive; Inh, Inheritance.

All samples were TE biopsies of 5–8 cells obtained from day 5 blastocysts. Written informed consent was obtained from all the participants (embryo donors). The ethics committees of the three IVF centers, (CEIC de LIDIAP *Jordi Gol i Gurina*, *Eugin*; CE *Investigació Clínica*, *Fundacio Puigvert* and CIMA, *Instituto Marques*), evaluated positively the objectives of the research project of this work according to "*Ley de reproducción humana asistida en España*”.

For the indirect diagnosis of monogenic disease by linkage analysis on embryos F2E1-GEH and F2E2-RG, genomic DNA samples from the affected father and grandmother and from the unaffected mother were also studied.

### Whole genome amplification (WGA)

DNA Products from four WGA systems were tested as template for TSO sequencing panel for mutation detection and CNV analysis. Pools containing 8 cells were amplified by using the following WGA kits: Sureplex (SurePlex DNA Amplification System, PR-40-415101-00, BlueGnome), MALBAC (One Single Cell WGA kit, Yikon Genomics; YK001B), GEH-MDA (illustra GenomiPhi V2 DNA Amplification, GE Healthcare; Kit Cat No./ID 25660030) and RG-MDA (REPLI-g Single Cell kit, Qiagen; Cat No./ID:150343). GEH-MDA is an MDA (Multiple Displacement Amplification) protocol adapted by Kumar *et al*. [25] The amplifications of DNA using RG-MDA, Sureplex, and MALBAC kits were performed according to the manufacturer’s instructions. To minimize the ADO incidence using GEH-MDA, a DMSO gradient (0%, 3%, 5% and 7%) was performed in GM03184 fibroblast cell line (Fig 1).

DNA quality and fragment size after WGA was monitored in 1.5% agarose gel electrophoresis.

To determine the applicability of the different WGA systems to TSO sequencing panel, TE biopsies from embryos F11E1-S, F12E1-M, F7E1-GEH and F13E1-RG were amplified by using Sureplex, MALBAC, GEH-MDA (without DMSO) and RG-MDA, respectively.

For the translational application of the NGS-TSO panel to DF-PGT, six embryos were amplified by using GEH-MDA amplification system with the addition of 5% DMSO and other six embryos were amplified by using RG-MDA amplification system (Table 1).

### Diagnosis of monogenic diseases by NGS

The Illumina TSO sequencing panel was analyzed on MiSeq and Nextseq 500 sequencers according to the manufacturer’s instructions (Illumina).

BaseSpace Sequence Hub (Illumina) was used for sequencing data analysis. Reads were aligned with Burrows-Wheeler Aligner (BWA) against the human reference genome hg19 (Homo sapiens, hg19, build 37.2). For genotype analysis VariantStudio software ver. 2.1.46 (Illumina) and Integrative Genomics Viewer software ver. 2.3.32 softwares were used. All regions with a sequencing depth <10X were considered to be unsuitable for analysis. The ADO rate was measured by the ratio of undetected heterozygous SNVs in amplified DNA compared with not amplified genomic DNA from GM03184 cell line. False positive rate (FPR) was measured considering the percentage of SNPs of the amplified DNA not present in genomic DNA from GM03184 cell line versus the total sequenced base pairs.

For the indirect detection of the allele carrying the family mutation, a linkage analysis was performed. Informative SNPs haplotypes (haploblocks) associated to the mutated and the normal allele were evaluated. To be considered informative, these SNPs had to be found in a heterozygous state in the carrier and in a homozygous state in the healthy parent. The study of the alternant segregation of heterozygous SNPs in affected and not affected family members will allow for identification of those SNPs linked to the mutation associated to the inheritance of the disease. In addition, when the heterozygous SNPs of an affected parent (homozygous in the healthy parent) has been inherited from a homozygous affected grandparent, a fully informative linkage to the disease can successfully be inferred. This linkage analysis was performed in family F2, in which the grandmother and the father were carriers of a mutations in *EXT1* and suffer from exostoses, while the mother was healthy, not carrier of the mutation.

### Copy number variation (CNV) determination by NGS

BAM files generated after sequencing following alignment against human genome were analyzed for CNVs using the Nexus Copy Number 8.0 Software (Bio Discovery). The log-ratio of signal-intensity values for DNA content at each measured locus was calculated and compared to the reference baseline. The software uses FASST2, which is an implemented algorithm that segments the log-ratio data and identifies copy number changes. Systematic correction was applied to avoid GC biases. The settings criteria used are included in S1 Table.

### CNV-NGS results versus metaphase CGH (mCGH) and oligonucleotide arrays CGH (aCGH)

The SurePrint G3 Human CGH Microarray Kit (Agilent) was used following the manufacturer's instructions. Agilent protocol for genomic DNA analysis was performed on a 4x180K array format, except that the 2h restriction digestion step was not performed. Workbench Standard Edition software (Agilent) was used for the cytogenetic analysis. The ADM-2 algorithm was applied for determining cytogenetic abnormalities. mCGH was performed as previously described.[26]. All twelve embryos included in the DF-PGT strategy were analyzed by mCGH. Embryos F1E2-RG, F2E2-RG, F3E2-RG and F8E2-RG were reanalyzed by aCGH.

## Results

### TSO sequencing panel outcome using the product of four different WGA systems

**Fig 2:**
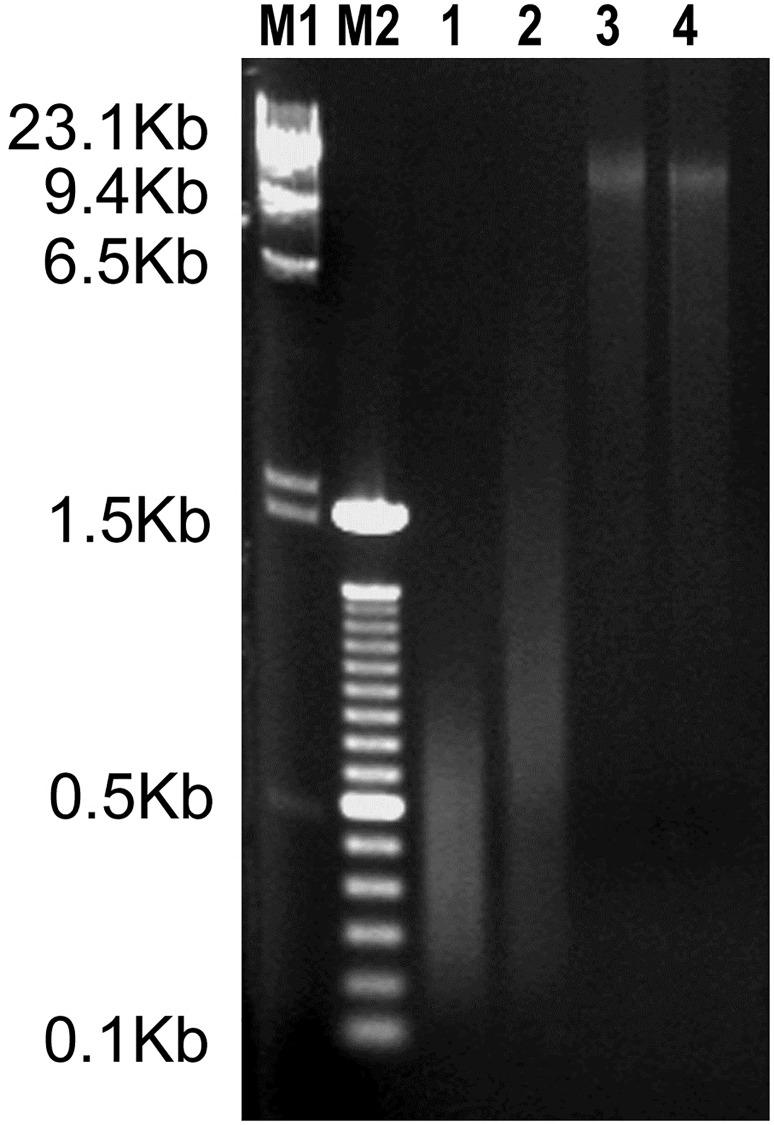
Gel analysis of WGA products from TE biopsies. M1, DNA molecular size marker; M2, DNA molecular size marker; Lane 1, MALBAC; Lane 2, Sureplex; Lane 3, GEH-MDA; Lane 4, RG-MDA.

To determine the applicability of the different WGA systems to the proposed DF-PGT protocol, four TE biopsies were satisfactorily amplified by using Sureplex, MALBAC, GEH-MDA and RG-MDA. The four WGA products were visualized in an agarose gel. Fragments’ size range was 200–2000 bp for Sureplex, 200–800 bp for MALBAC and 2->10 kb for the two MDA methods (GEH-MDA and RG-MDA). All obtained DNA products exhibited good quality, with the expected DNA amount and size. The two MDA DNA products showed the largest DNA fragments (Fig 2).

**Fig 3:**
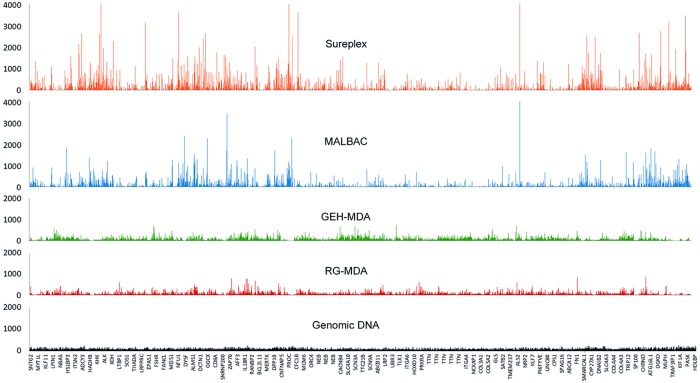
Graphical representation of the variation along the chromosome 2, of depth uniformity coverage parameter in the WGA DNA systems used A) MALBAC, B) Sureplex, C) GEH-MDA, D) RG-MDA and in E) not amplified genomic DNA.

Regarding the evaluation of coverage and depth uniformity, after TSO, Sureplex and MALBAC presented both large uncovered regions and extremely overrepresented regions. GEH-MDA and RG-MDA methodologies showed a homogenous coverage along the genome, similar to that observed for genomic DNA. When comparing the two MDA based methods, RG-MDA presented a better genome coverage than GEH-MDA (Fig 3). Regarding percentage of regions with a coverage >10X, results showed that the RG-MDA method was the most efficient, with almost 95% of the regions having at least 10X depth, followed by GEH-MDA, Sureplex and MALBAC amplification protocol, with 85%, 65.5% and 64%, respectively. According to these results, both MDA-based methods were considered suitable to generate the DNA template for TSO sequencing panel, and MALBAC and Sureplex protocols were excluded from further analyses with TSO.

#### Evaluation of ADO and coverage with MDA-based WGA methods in GM03184 cell line

**Fig 4:**
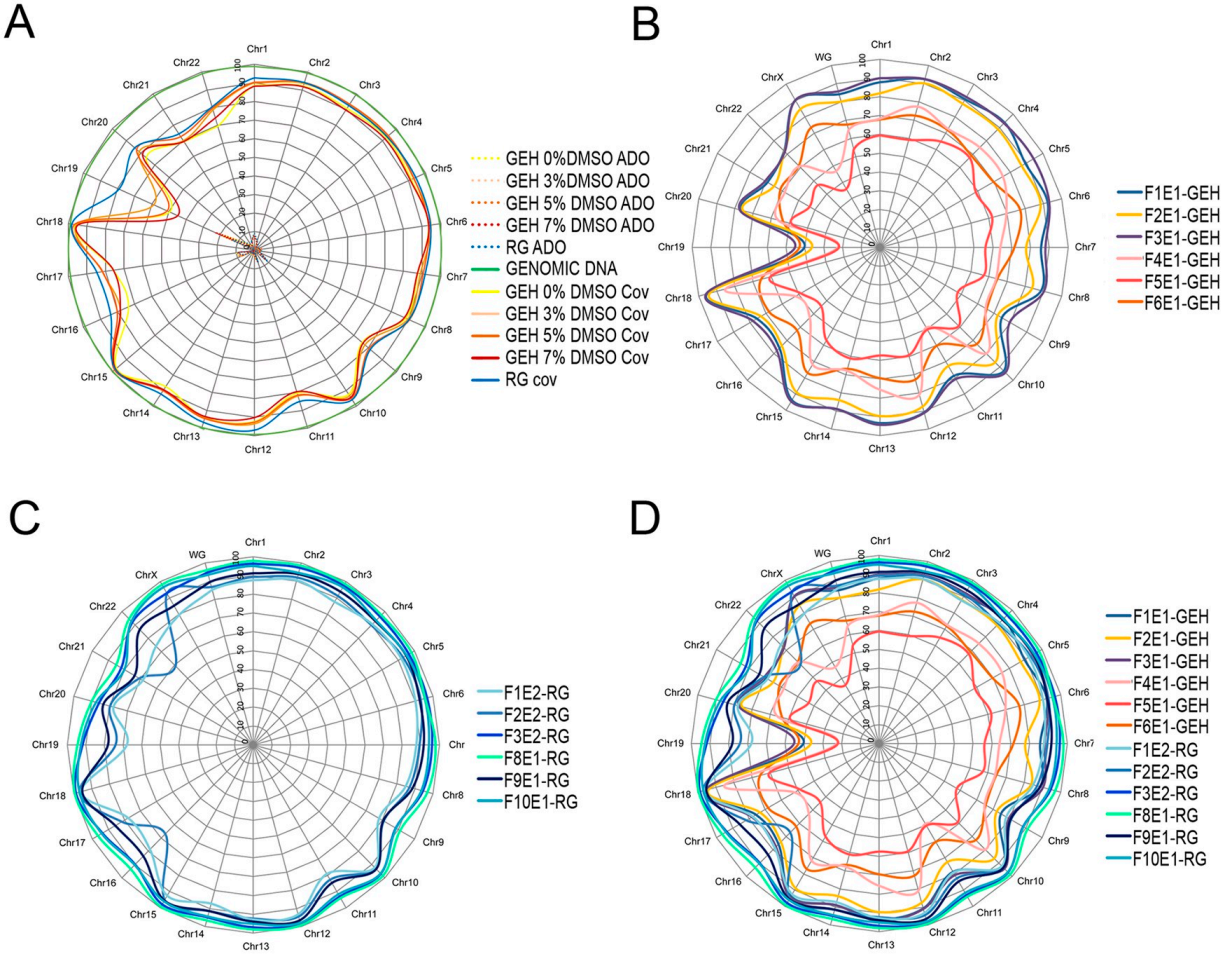
Graphical representation of TSO coverage at ≥10X by chromosome after DNA amplification with different WGA methods. A) Coverage and ADO rate of GM03184 Coriel cell line WGA products (GEH-MDA 0% DMSO, GEH-MDA 3% DMSO, GEH-MDA 5% DMSO, GEH-MDA 7% DMSO and RG-MDA) and genomic DNA; B) Coverage of GEH-MDA 5% DMSO WGA products from embryos; C) Coverage of RG-MDA WGA products from embryos; D) Comparison of the coverage of all the WGA products from embryos. RG-MDA displayed the best chromosome coverage.

For the quantification of ADO incidence from MDA-based methods, both genomic DNA and WGA products from the GM03184 cell line were processed and analyzed with TSO panel. All processed MDA products (including GEH-MDA with and without DMSO and RG-MDA without DMSO) displayed the expected fragment size range mentioned before and with no apparent DMSO effect on the GEH-MDA reaction. Coverage at ≥10X depth was 97.2% in genomic DNA and between 84.6 and 86.8% in GEH-MDA from the cell line sample. In this case, addition of 3% and 5% DMSO improved coverage observed on the GEH-MDA samples. Best coverage was observed in RG-MDA, with a value of 89.7% (Table 2). Poor coverage was observed in Chromosomes 16, 17, 19, 21 and 22 (Fig 4A). It is worth mentioning the substantial coverage increase observed when using RG-MDA, which was especially evident for chromosome 19 (Fig 4).

**Table 2:** Coverage at ≥10X, allele dropout, lack of amplification and false positive rate NGS-TSO results in WGA GM03184 Coriell cell line.

Sample	Coverage (%)	ADO(%)	LA(%)	Detected heterozygous loci	FPR
GEH-MDA 0% DMSO	84.6	8.5	13.51	3684	0.90x10^-5^
GEH-MDA 3% DMSO	86.2	11.2	9.96	3663	1.67 x10^-5^
GEH-MDA 5% DMSO	86.8	9.3	9.91	3743	1.63 x10^-5^
GEH-MDA 7% DMSO	84.6	7.5	11.42	3752	1.09 x10^-5^
RG-MDA	89.7	9	8.38	3868	1.16 x10^-5^

ADO, allele dropout; LA; lack of amplification of both alleles; FPR, false positive rate

The ADO values varied between 7.5% and 11.2% in samples of the cell line GM03184 amplified with GEH-MDA. The addition of DMSO to the DNA amplification reaction resulted in a better coverage and consequently a greater detection of heterozygous loci (reducing the sum of the ADO plus LA) (Table 2). RG-MDA was the WGA system that detected a greater number of heterozygous loci, presenting an ADO value of 9. The location of most of the ADO events correlated with the regions with low coverage (Fig 4A). In all the sample FPR values were between 0.9x10^-5^ and 1.6× 10^−5^. No significant additional DNA amplification errors were generated by DMSO supplementation (Table 2).

### Cytogenetic analyses of GM03184 cell line by mCGH

All MDA products were evaluated by using mCGH in order to verify the representativeness of WGA products respect to the original genome of an aneuploid (+ 15) GM03184 cell line. All tested conditions for MDA-based systems (GEH-MDA with 0%, 3%, 5%, 7% DMSO and RG-MDA) allowed for the proper detection of chromosome 15 gain by mCGH. A negative correlation between the quality of mCGH profile and the addition of DMSO to the GEH-MDA protocol was found. No extra alterations were found in all samples, except for that with highest concentration of DMSO (7%), which displayed numerous mCGH artifactual imbalances. So, the further analyses on TE biopsies by using TSO were performed with GEH-MDA with a 5% DMSO and RG-MDA since were the methods which offered the best results.

### Translational applicability of the NGS-TSO panel to DF-PGT: PGT-M of the embryos

Based on the previous results, the proposed DF-PGT strategy was applied to 12 TE biopsies from 12 different PGT-M discarded embryos donated by nine families at risk of having offspring affected by a monogenic disease (Table 1). Six TE biopsies were amplified by using GEH-MDA with 5% DMSO and six by using RG-MDA. Regarding genome coverage, RG-MDA provided again better coverage than GEH-MDA with 5% DMSO (Fig 4B–4D and Table 3). This improvement was even more evident in embryos than in the previous setup experiments with GM03184 cell line. RG-MDA amplification from TE biopsies also displayed better chromosome 19 coverage compared to GEH-MDA results (Fig 4D).

**Table 3:** Percentage of genome coverage at ≥10X of the GEH WGA of embryos from the F1, F2, F3, F4, F5 and F6 families and of the WGA RG of embryos from the F1, F2, F3, F8, F9 and F10 families.

Sample	Coverage (%)
F1E1-GEH	83.9%
F2E1-GEH	80.1%
F3E1-GEH	85.7%
F4E1-GEH	65.6%
F5E1-GEH	56.0%
F6E1-GEH	67.8%
F1E2-RG	84.8%
F2E2-RG	86.2%
F3E2-RG	93.6%
F8E1-RG	95.7%
F9E1-RG	89.2%
F10E1-RG	92.7%

Results of direct mutation diagnosis of the embryos amplified by RG-MDA showed that five out of six embryos previously diagnosed as affected were properly and concordantly diagnosed by NGS (Table 4). The other embryo (F2E2-RG), which had not been diagnosed during PGT-M for an *EXT1* mutation was found to be mutation free by NGS-TSO. This was later confirmed by Sanger sequencing and by linkage analysis with informative SNPs from the same TSO sequencing data.

**Table 4:** Mutation detection and cytogenetic results of the GEH-MDA WGA of embryos from the F1, F2, F3, F4, F5 and F6 families and of the RG-MDA WGA of embryos from the F1, F2, F3, F8, F9 and F10 families by NGS.

Embryo	Gene	Mutation	Mutation detection by NGS-TSO	CNV detection with mCGH	CNV detection with NGS
F1E1-GEH	*PTCH1*	c.2776delT	Detected	Chaotic	-5q11q31.3, -11p
F2E1-GEH	*EXT1*	c.1468delC	Detected*	Chaotic	+3q, -5p, +19q
F3E1-GEH	*BRCA1*	c.68_69delAG	Detected	Euploid	-4p11p15.33, -9q
F4E1-GEH	*ATXN3*	c.873_874CAG[70]	Not detected	Chaotic	-1q, +2p, +4q, +5q, +7p, +8p, +9, +10q, -11p, +12q, -16p, -17p, -19q, -20, -Xq
F5E1-GEH	*PKLR*	c.721G>T	Detected	Chaotic	+3, +7p, -9p, +14, -18q, -20p, +20q, +22, -Xp
		c.204delC	Not detected		
F6E1-GEH	*FZD4*	c.1501_1502delCT	Not detected	Chaotic	-3q, -4p, -5p, +6p, +7q, -10p, +10q, +19q, -20p, -22, +X
F1E2-RG	*PTCH1*	c.2776delT	Detected	Euploid	Euploid
F2E2-RG	*EXT1*	c.1468delC	Healthy*	-16, -22	-16, -22
F3E2-RG	*BRCA1*	c.68_69delAG	Detected	+11pter11q13, -11q23qter	+11pter11q13, -11q23qter
F8E1-RG	*MLH1*	c.199G>T	Detected	-6	-6
F9E1-RG	*APC*	c.4611_4612delAG	Detected	Euploid	Euploid
F10E1-RG	*APC*	c.1660C>T	Detected	-2q21.1qter, +19, +20p	-2q24.3q35, -15q11q21.2, +19p, +20p

* Diagnosed by direct mutation detection and indirect mutation detection by SNP linkage analysis

**Fig 5:**
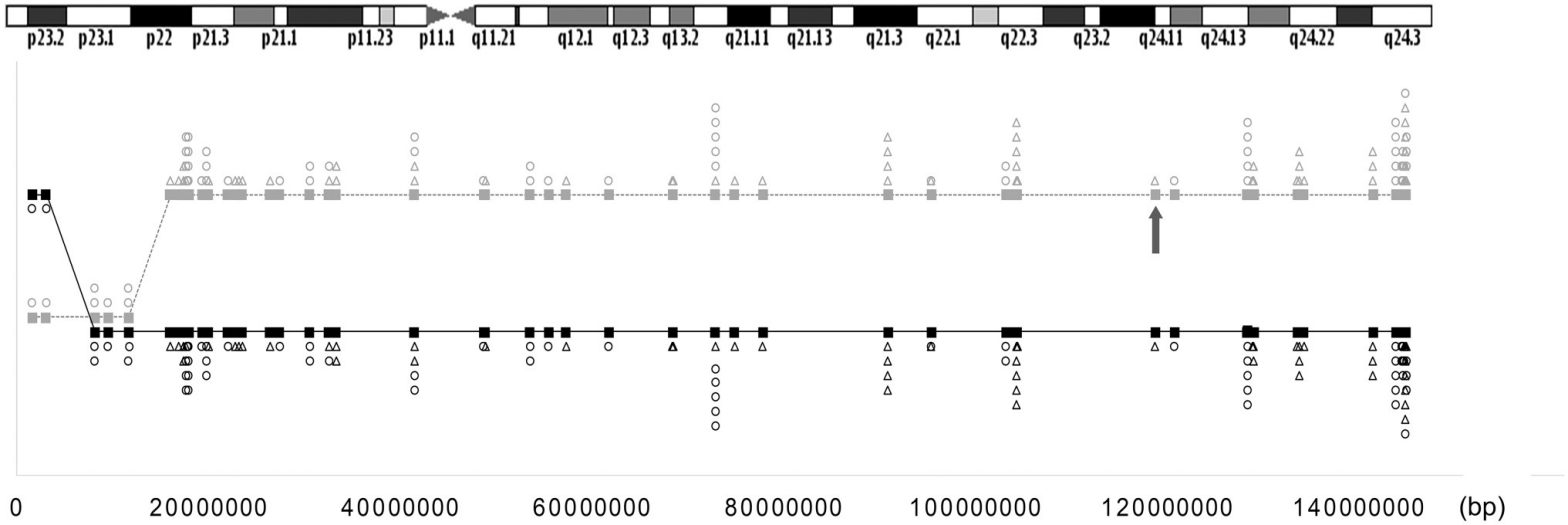
Representation of paternal heterozygous SNPs of chromosome 8 and its segregation in embryos F2E2-RG (healthy) (solid black line) and F2E1-GEH (affected by exostosis) (dashed gray line). Of the informative SNPs inherited from the father in each of the two embryos, the SNPs alternately inherited are represented with a triangle while the segregation of the 100% informative SNPs are represented with a circle. Mutation is represented with an arrow.

The indirect mutation diagnosis by linkage was performed studying the heterozygous SNPs of the father and homozygous in the mother in embryo F2E1-GEH. This allowed the identification of an haploblock of 127 Mb associated to the disease. The absence of a mutant allele in F2E2-RG embryo plus the alternant segregation of 108 SNPs surrounding *EXT1* mutation in chromosome 8 (54 of them were fully informative being homozygous in affected grandmother, so associated to the disease), supports that each embryo (F2E1-GEH and F2E2-RG) from family F2 have received a different haploblock from the affected father. F2E1-GEH received the mutant allele, while F2E2-RG received the healthy allele. (Fig 5 and Table 4).

### Translational applicability of the NGS-TSO panel to DF-PGT: PGT-A of the embryos

Regarding the PGT-A results of the TE biopsies performed by using GEH-MDA amplification products, five out of the six embryos displayed completely discordant mCGH and NGS-CNV results. Both analyses resulted in multiple aneuploidies and segmental imbalances. NGS-CNV displayed widely scattered profiles. The remaining sample, F3E1-GEH, showed a partially concordant result between NGS-CNV and mCGH: it was diagnosed as euploid by mCGH, but a loss of 4p11p15.33 and 9q chromosome was observed by NGS-CNV (Table 1) (see S1–S6 Figs).

**Fig 6:**
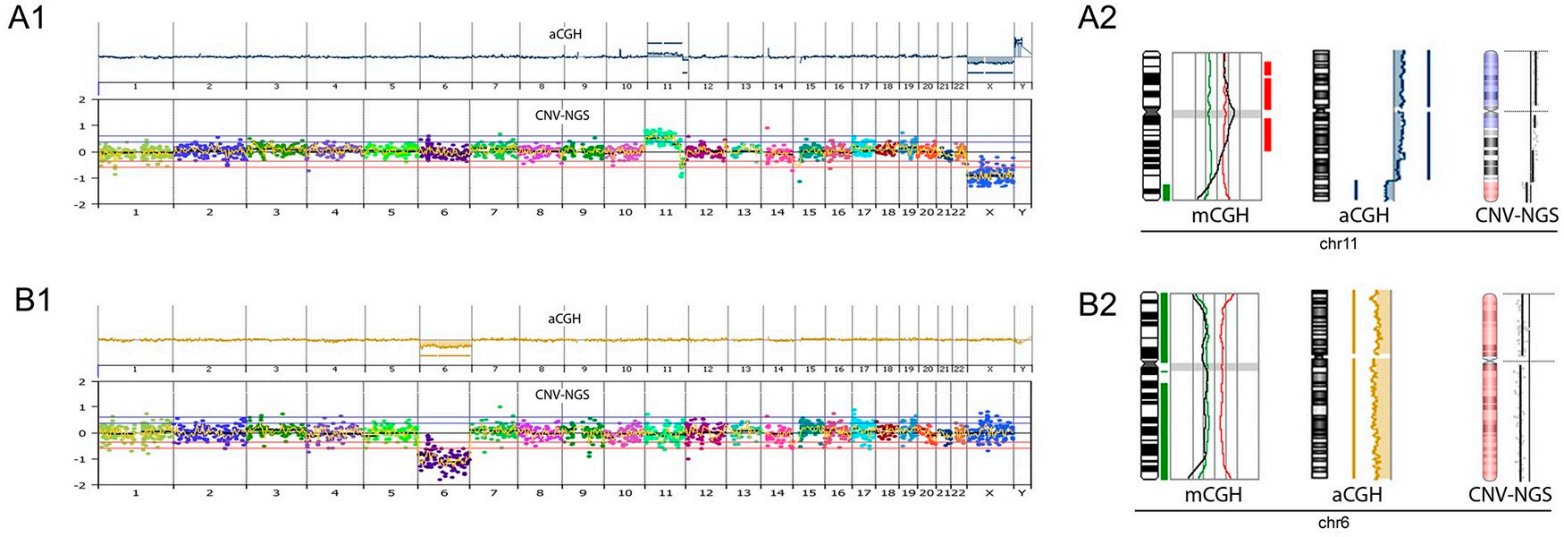
A-B) Examples of totally coincident results with the three cytogenetic analysis platforms used in A) embryos F3E2-RG and B). F8E1-RG. A1 and B1 show the whole chromosome complement with aCGH (top) and NGS-CNV (bottom) and A2 and B2 show the chromosome imbalance detected for each embryo by using mCGH, aCGH and CNV-NGS.

The cytogenetic analysis of TE biopsies from six embryos was performed by using RG-MDA amplification products. Results of these samples showed good quality profiles without dispersion and concordance between mCGH and NGS-CNV protocols in five of them (S7–S11 Figs). In addition, the NGS approach was able to detect chromosomal reorganizations such as that found in chromosome 11 of F3E2-RG sample, later confirmed by mCGH and aCGH (Fig 6). Only a partially discordant result, affecting chromosomes 15q and 19q, was found in embryo F10E1-RG (Table 4). Both mCGH and NGS-CNV showed dispersed profiles. (S12 Fig).

## Discussion

One of the main objectives of the present study was to develop a new diagnostic tool that increases the implantation rate in the Preimplantation Genetic Testing (PGT) cycles. With this aim some Double Factor PGT strategies have been successful used. [9,27] The most viable healthy embryos diagnosed in PGT could be selected for transfer when the family mutation analysis is performed in addition to the 23 chromosomes, through 1^st^ Polar Body-mCGH or through Blastomere-mCGH analysis. But most of the PGT requires the implementation of the direct and indirect diagnostic procedures specifics for each family mutation. Karyomapping, described by Handyside et al., (2010), overcomes this handicap and involves a substantial improvement in PGT.[15,28]. Karyomapping is based on the identification SNPs and characterization of parental haplotypes in embryos. Applied to PGT, the analysis of WGA products from embryo samples but also of the study of parental and other family members of interest to perform linkage mutation analysis for the specific family mutation is needed. No previous set up step is required for each family mutation. Moreover, a 24Chr CNV analysis can be performed. The detection of the CNV made by Karyomapping is also based on the identification of parental haplotypes in the embryos. Monosomies or deletions, whether of meiotic or mitotic origin, can be identified by the lack of one of the chromosomal parental haplotypes. Meiotic trisomies, as a consequence of the inheritance of both chromosomes of a parent, are identified by the presence of both haplotypes in the embryo. However, the post-zygotic chromosomal duplications, consequence of mitotic segregation errors of sister chromatids are not detected by Karyomapping, since the sequences of both chromosomes are identical.

The implementation of high performance diagnostic NGS platforms to the PGT, such as TSO panel, that includes the thousands of genes responsible for the most frequent genetic diseases in the population and that also allows the identification of cytogenetic, is a proposal of great interest. In addition, the previously mentioned limitations are overcome: no particular set up for the direct and indirect mutation detection is needed and allowing for de detection of any cytogenetic imbalance independently from their origin.

In fact, Yan et al., 2015 have published a DF-PGT strategy using a NGS-based PGT procedure that can simultaneously detect a single-gene disorder and aneuploidy. The MARSALA strategy “Mutated Allele Revealed by Sequencing with Aneuploidy and Linkage Analyses” combines next-generation sequencing and single-cell whole-genome amplification methodologies. But, this approach requires to adapt the protocol to each family mutation.[21]

In the present study, is described the development and optimization of a new procedure suitable for the simultaneous detection of both disease-causing mutations and chromosomal abnormalities (DF-PGT). Experimental proceedings of the set up work have been performed on pools of eight cells, hereby simulating TE biopsies. Several works have been conducted to validate CNV detection by NGS on TE biopsies using different WGA systems.[29–31] Also, Sureplex amplification product from single blastomere has been recently applied. [29,32] Regarding RG-MDA, a study of Huang *et al*., suggested that RG-MDA is prone to generate more amplification bias and artifacts than Sureplex or MALBAC, but this study was performed on single-cell amplification product.[24] However, RG-MDA products from TE biopsies were successfully applied to PGT by using Karyomapping, allowing for the detection of a *de-novo* deletion undetectable by conventional PGT.[16]

For the first step of the present work, evaluate the applicability of the different WGA products to TSO panel, four different commercially available WGA systems (Sureplex, MALBAC, GEH-MDA and RG-MDA) were tested. Previous works have been described a different genetic diagnostic performance in cell lines and embryos. This phenomenon has been attributed to the intrinsic characteristics of the cell type analyzed (blastomere versus cell lines), rather than to the amplification methodology used. [10] For this reason, the set up step has been performed with trophoectoderm samples. The obtained size range of fragments and amount of DNA for each WGA system were concordant with previously published data. [23,33,34] As expected, the size range of MDA products has been shown to be larger than other WGA commercial methods (Fig 2). At the same time, it has been seen that the DNA size range and amplification amount product was not affected by the addition of DMSO in GEH-MDA reaction. On the other hand, DNA amplified with Sureplex and MALBAC systems, which are widely used in PGT, displayed poor performances as a template for NGS-TSO panel. The reason for this failure could be explained by the size of the fragments generated by these two WGA system (see Fig 2). Despite all four WGA techniques are known to generate DNA amplification products faithful to the original template, only MDA systems produce large enough DNA fragments. The first step of NGS library preparation requires both DNA fragmentation and adaptor incorporation by a transposase. Starting from a wide-range of low molecular weight material, such as the amplified DNA products from Sureplex and MALBAC protocols, could compromise the accuracy of the NGS protocol performance. The smear of short fragments of Sureplex and MALBAC could generate the observed loss of uniformity in depth coverage along the whole genome in the TSO sequencing (Fig 3). Nevertheless, this lack of homogeneity of depth uniformity of coverage, observed in TSO, may not be observed in other sequencing methodologies not limited by the size of the DNA. [21]

A previous study had tested RG-MDA performance on single-cell amplification for exome sequencing, but with opposite results to those shown in this work.[23] This can be explained by the fact that MDA includes several different commercial kits, each one specifically addressed to different input material, unlike what happens with Sureplex and MALBAC systems. While RG-MDA mini kit needs an input of >10ng DNA sample, the RG-MDA system used in the present work is adequate (according to the manufacturer’s specifications) to the analyses of DNA from single cells.

Regarding to the one step DF-PGT by using NGS strategy, this work allowed the identification of a WGA system very convenient for the further processing and analysis with NGS-TSO panel. Since more than 4,800 genes are expected to be sequenced, WGA products must be faithful to the original genome and long enough to not compromise the amplification protocol. Both GEH-MDA and RG-MDA WGA methods demonstrated a low error rate after NGS-TSO analysis, very similar to other gold-standard WGA methods widely used in the PGT and other scientific fields such as bacterial genome amplification (Table 2).[24,35]

It is well known that GC-rich sequences exhibit problematic and biased PCR DNA amplification. Addition of DMSO to the GEH-MDA reaction mix favors amplification of these regions, reducing ADO rate.[36] In agreement with previous studies, results of the present work with NGS-TSO highlighted that ADO incidence is more evident in genes located on clear band chromosomes (especially on chromosomes 16, 17 and 19) (Fig 4A). When using mCGH and aCGH to analyze embryos, these chromosomes usually present artifacts regions.[14,33] A positive correlation between DMSO addition and ADO rate reduction in the GEH-MDA method has been demonstrated in this work (Table 3). Moreover, results also showed that the RG-MDA method produces less ADO and better coverage than any experimental condition tested for GEH-MDA.

Regarding to the sensitivity of mutation detection tested in embryos diagnosed from GEH-MDA with 5% DMSO amplification products, four out of the seven mutations were properly detected (Table 4). Two out of the three embryos in which the mutation was not detected, F5E1-GEH and F6E1-GEH, showed low coverage of sequence at the corresponding genes (*PKLR1* and *FZD4* respectively). For the third failed diagnosis (F4E1-GEH), the causative mutation corresponds to a trinucleotide CAG(n) repeat expansion in *ATXN3*. It is noteworthy that NGS procedures do not properly detect the variation in repetitive polymorphic,[37] so TSO is not addressed to the diagnose illnesses that are consequence of triplet expansions.

Direct mutation detection with RG-MDA products was successfully achieved using the TSO panel in all tested embryos. All family mutations were located in well covered loci. The embryo undiagnosed for the familial *EXT1* mutation in a previous PGT-M cycle, was found to carry the healthy allele. The absence of this mutation was confirmed by SNP linkage analysis, discarding the possibility of an ADO of the mutated allele. Thus, the proposed methodology allows direct detection of the causative mutation and concomitant indirect linkage analysis without any previous set up step, an advantage over traditional methods that require a laborious previous work of informative markers selection. This methodological approach has some equivalences with Karyomapping, as both are based on informative SNP analysis. However, Karyomapping strategy uses a higher number of SNP markers because it includes intronic SNPs[38], while TSO only evaluates exonic regions. Nevertheless, Karyomapping approach in TE embryo analysis resulted in a 10% of inconclusive results.[15] The present strategy has been successfully applied for the indirect detection of a paternal mutation. It is interesting to point that male meiosis displays lower number of recombination points than females. According to this, Codina et al. found that chromosome 8 in male meiosis presents a median of two recombination points per chromosome (range 0–2, in the arm p and q) that is concordant with the fact that only one recombinant point at p-arm has been identified.[39] The potential applicability of linkage tracking of mutations of maternal origin could not be discarded. In addition, larger target enrichment panels, such as whole exome sequencing will allow the analysis of more genes and therefore more exonic SNPs, which would represent a very promising future for this strategy. In this sense, it would be possible the enrichment of the sequencing panels with more informative SNPs through the addition of intronic regions. This would improve the strategy and result in a robust DF-PGT application. However, the most indisputable advantage of the present strategy compared to Karyomapping is the fact that it allows for the direct detection of the familial mutation concomitantly with SNP linkage analysis. As far as we know, it is the first time that a target enrichments gene panel (like TSO) has been successfully applied for the direct and indirect detection of monogenetic disease and CNV analysis on embryo biopsies. CNV-NGS artifacts involving clear band chromosomes have been observed. These bands correspond to GC-rich regions and consequently prone to generate artifacts, as has been previously described by mCGH in chromosomes 1p, 16, 17, 19 and 22.[14,26]

This work demonstrates that the addition of 5% DMSO in GEH-MDA reaction leads to a significant reduction of ADO rates in TSO outcome and better coverage uniformity with no adverse effects on mCGH analysis in Coriell cell lines. However, the addition of 5% DMSO in GEH-MDA WGA generated artifacts both for mCGH and CNV-NGS in embryos (Table 4). Reasons for the artifact generation could be related to the manual processing of the samples, since the embryo transfer to the PCR tube is manually performed making difficult to adjust the final reaction volume.

Altogether and despite the reduction observed in the ADO rate with the addition of 5% DMSO, the discrepancies noticed between mCGH and NGS-TSO in TE biopsies analyzed would not recommend the application of GEH-MDA approach to the DF-PGT.

To our knowledge, this is the first time that it has been successfully inferred the cytogenetic characterization of RG-MDA WGA product from TE biopsies, by using TSO panel data. Nexus Copy Number software was satisfactory used for the analysis of RG-MDA and results were coincident between the three techniques used: mCGH and aCGH and also in CNV-TSO. Only two segmental discrepancies were detected affecting 15q and 19q, (in F10E1-RG sample). This only affects two chromosomes of one of the seven biopsies (two out of 168 analyzed chromosomes) (Table 4). Some discordances have been observed in previous studies between NGS and aCGH when two TE biopsies with the same or different WGA system had been analyzed. Such discrepancies can be a consequence of mosaicism, WGA bias or expected inherent lack of concordance between whole chromosome analysis technologies.[19,30] In the present work, since the analyzed DNA samples are aliquots from a single biopsy amplification product, mosaicism and WGA bias can be discarded. Moreover, it has been reported that the rate of false positives when applying aCGH to TE biopsies is approximately 9%.[40]

We also demonstrated that data from NGS-TSO allows not only the accurate aneuploidy detection but also the identification of unbalanced chromosome reorganizations, such as the chromosome 11 reorganization (+11pterq13, -11q23qter) that was found in the F3E2-RG sample. This reorganization was confirmed by mCGH and aCGH. In summary, the results obtained by Nexus Copy Number software with TSO data, nowadays the most comprehensive gene panel of Mendelian inheritance diseases is highly satisfactory to the CNV studies in WGA products from TE biopsies. Regarding the cytogenetic applicability, compared to Karyomapping, the approach used in this work identifies whole chromosome or segmental imbalances independently from their origin (mitotic or meiotic) in a single experimental procedure analyzing TSO data by using Nexus Copy Number software by a properly trained geneticist. In sum, despite the satisfactory and very promising results of the present study regarding to the cytogenetic characterization of cell lines and embryos, additional studies should be performed before using this approach as a diagnostic alternative.

Blastomere biopsy versus trophectoderm biopsy has been reported to result into a 19% absolute reduction and 39% relative reduction of the implantation rate.[41]. The whole procedure can be performed in about six days in both fresh and cryopreserved TE biopsies. The samples analyzed by NextSeq 500 instead of MiSeq sequencer, due to it allows for the analysis of higher number of samples, leads to a significant cost reduction. The present cost per sample is considerable (300€/sample). The advent of new sequencers, the automation of the procedure and altogether with the cheapening of the reagents will promote a substantial reduction of overall cost per sample.

In conclusion, the present work offers a powerful DF-PGT strategy that enables diagnoses of both monogenic causative mutations and cytogenetic alterations of blastocyst stage embryos in PGT candidate families, which is a very universal pursuit goal among different research groups. As for the monogenic mutation detection, the direct detection of the familial mutation is allowed concomitantly with SNP linkage analysis. The development of larger target enrichment panels, such as whole exome sequencing, will allow the analysis of more genes and therefore more SNPs, which would represent a very promising future for this strategy. As far we know, this is the first time that a potentially suitable DF-PGT approach based on a NGS strategy in order to be performed in a single workflow has been successfully developed.

## Supporting information

S1 Table Nexus software settings for CNV analysis. (DOCX)

S1 Fig Embryo F1E1-GEH. On the left, mCGH results, reference used 46, XY. On the right, NGS-CNV summary plot, reference used 47,XY+15. Note that the loss of chromosome 15 observed is due to the reference used.
(TIF)

S2 Fig Embryo F2E1-GEH. On the left, mCGH results, reference used 46, XY. On the right, NGS-CNV summary plot, reference used 47,XY+15. Note that the loss of chromosome 15 observed is due to the reference used.
(TIF)

S3 Fig Embryo F3E1-GEH. On the left, mCGH results, reference used 46, XY. On the right, NGS-CNV summary plot, reference used 47,XY+15. Note that the loss of chromosome 15 observed is due to the reference used.
(TIF)

S4 Fig Embryo F4E1-GEH. On the left, mCGH results, reference used 46, XY. On the right, NGS-CNV summary plot, reference used 47,XY+15. Note that the loss of chromosome 15 observed is due to the reference used.
(TIF)

S5 Fig Embryo F5E1-GEH. On the left, mCGH results, reference used 46, XY. On the right, NGS-CNV summary plot, reference used 47,XY+15. Note that the loss of chromosome 15 observed is due to the reference used.
(TIF)

S6 Fig Embryo F6E1-GEH. On the left, mCGH results, reference used 46, XY. On the right, NGS-CNV summary plot, reference used 47,XY+15. Note that the loss of chromosome 15 observed is due to the reference used.
(TIF)

S7 Fig Embryo F1E2-RG. On the left, mCGH results, reference used 46, XY. On the right, NGS-CNV summary plot, reference used 46,XX.
(TIF)

S8 Fig Embryo F2E2-RG. On the left, mCGH results, reference used 46, XY. On the right, NGS-CNV summary plot, reference used 46,XX.
(TIF)

S9 Fig Embryo F3E2-RG. On the left, mCGH results, reference used 46, XY. On the right, NGS-CNV summary plot, reference used 46,XX.
(TIF)

S10 Fig Embryo F8E1-RG. On the left, mCGH results, reference used 46, XY. On the right, NGS-CNV summary plot, reference used 46,XX.
(TIF)

S11 Fig Embryo F9E1-RG. On the left, mCGH results, reference used 46, XY. On the right, NGS-CNV summary plot, reference used 46,XX.
(TIF)

S12 Fig Embryo F10E1-RG. On the left, mCGH results, reference used 46, XY. On the right, NGS-CNV summary plot, reference used 46,XX.
(TIF)

## References

[1] De Rycke M, Belva F, Goossens V, Moutou C, SenGupta SB, Traeger-Synodinos J, et al ESHRE PGD Consortium data collection XIII: cycles from January to December 2010 with pregnancy follow-up to October 2011. Hum Reprod. 2015;30: 1763–89. 10.1093/humrep/dev122 26071418

[2] Voullaire L, Wilton L, McBain J, Callaghan T, Williamson R. Chromosome abnormalities identified by comparative genomic hybridization in embryos from women with repeated implantation failure. Mol Hum Reprod. 2002;8: 1035–41. Available: http://www.ncbi.nlm.nih.gov/pubmed/12397217 12397217 10.1093/molehr/8.11.1035

[3] Rubio C, Simón C, Vidal F, Rodrigo L, Pehlivan T, Remohí J, et al Chromosomal abnormalities and embryo development in recurrent miscarriage couples. Hum Reprod. 2003;18: 182–8. Available: http://www.ncbi.nlm.nih.gov/pubmed/12525464 12525464 10.1093/humrep/deg015

[4] Fragouli E, Katz-Jaffe M, Alfarawati S, Stevens J, Colls P, Goodall N, et al Comprehensive chromosome screening of polar bodies and blastocysts from couples experiencing repeated implantation failure. Fertil Steril. 2010;94: 875–87. 10.1016/j.fertnstert.2009.04.053 19540479

[5] Baart EB, Martini E, van den Berg I, Macklon NS, Galjaard R-JH, Fauser BCJM, et al Preimplantation genetic screening reveals a high incidence of aneuploidy and mosaicism in embryos from young women undergoing IVF. Hum Reprod. 2006;21: 223–33. 10.1093/humrep/dei291 16155075

[6] Harton GL, Munné S, Surrey M, Grifo J, Kaplan B, McCulloh DH, et al Diminished effect of maternal age on implantation after preimplantation genetic diagnosis with array comparative genomic hybridization. Fertil Steril. 2013;100: 1695–703. 10.1016/j.fertnstert.2013.07.2002 24034939

[7] Obradors A, Rius M, Cuzzi J, Daina G, Gutiérrez-Mateo C, Pujol A, et al Errors at mitotic segregation early in oogenesis and at first meiotic division in oocytes from donor females: comparative genomic hybridization analyses in metaphase II oocytes and their first polar body. Fertil Steril. 2010;93: 675–9. 10.1016/j.fertnstert.2009.08.050 19878936

[8] Daina G, Ramos L, Rius M, Obradors A, del Rey J, Giralt M, et al Non-meiotic chromosome instability in human immature oocytes. Eur J Hum Genet. 2014;22: 202–207. 10.1038/ejhg.2013.106 23695274 PMC3895626

[9] Obradors A, Fernández E, Oliver-Bonet M, Rius M, de la Fuente A, Wells D, et al Birth of a healthy boy after a double factor PGD in a couple carrying a genetic disease and at risk for aneuploidy: case report. Hum Reprod. 2008;23: 1949–56. 10.1093/humrep/den201 18523000

[10] Daina G, Ramos L, Obradors A, Rius M, del Rey J, Martinez-Pasarell O, et al Double-factor preimplantation genetic diagnosis: Monogenic and cytogenetic diagnoses analyzing a single blastomere. Prenat Diagn. 2015;35: 1301–1307. 10.1002/pd.4691 26389801

[11] Treff NR, Zimmerman RS. Advances in Preimplantation Genetic Testing for Monogenic Disease and Aneuploidy. Annu Rev Genomics Hum Genet. 2017;18: annurev-genom-091416-035508. 10.1146/annurev-genom-091416-035508 28498723

[12] Natesan SA, Handyside AH, Thornhill AR, Ottolini CS, Sage K, Summers MC, et al Live birth after PGD with confirmation by a comprehensive approach (karyomapping) for simultaneous detection of monogenic and chromosomal disorders. Reprod Biomed Online. 2014;29: 600–605. 10.1016/j.rbmo.2014.07.007 25154779

[13] Rius M, Obradors A, Daina G, Ramos L, Pujol A, Martínez-Passarell O, et al Detection of unbalanced chromosome segregations in preimplantation genetic diagnosis of translocations by short comparative genomic hibridization. Fertil Steril. 2011;96: 134–42. 10.1016/j.fertnstert.2011.04.052 21596375

[14] Ramos L, del Rey J, Daina G, García-Aragonés M, Armengol L, Fernandez-Encinas A, et al Oligonucleotide arrays vs. metaphase-comparative genomic hybridisation and BAC arrays for single-cell analysis: first applications to preimplantation genetic diagnosis for Robertsonian translocation carriers. PLoS One. 2014;9: e113223 10.1371/journal.pone.0113223 25415307 PMC4240610

[15] Konstantinidis M, Prates R, Goodall N-N, Fischer J, Tecson V, Lemma T, et al Live births following Karyomapping of human blastocysts: experience from clinical application of the method. Reprod Biomed Online. 2015;31: 394–403. 10.1016/j.rbmo.2015.05.018 26206283

[16] Giménez C, Sarasa J, Arjona C, Vilamajó E, Martínez-Pasarell O, Wheeler K, et al Karyomapping allows preimplantation genetic diagnosis of a de-novo deletion undetectable using conventional PGD technology. Reprod Biomed Online. 2015;31: 770–5. 10.1016/j.rbmo.2015.08.017 26507283

[17] Treff NR, Fedick A, Tao X, Devkota B, Taylor D, Scott RT. Evaluation of targeted next-generation sequencing–based preimplantation genetic diagnosis of monogenic disease. Fertil Steril. 2013;99: 1377–1384.e6. 10.1016/j.fertnstert.2012.12.018 23312231

[18] Peters BA, Kermani BG, Alferov O, Agarwal MR, McElwain MA, Gulbahce N, et al Detection and phasing of single base de novo mutations in biopsies from human in vitro fertilized embryos by advanced whole-genome sequencing. Genome Res. Cold Spring Harbor Laboratory Press; 2015;25: 426–34. 10.1101/gr.181255.114 25672852 PMC4352880

[19] Kung A, Munné S, Bankowski B, Coates A, Wells D. Validation of next-generation sequencing for comprehensive chromosome screening of embryos. Reprod Biomed Online. 2015;31: 760–769. 10.1016/j.rbmo.2015.09.002 26520420

[20] Deleye L, De Coninck D, Christodoulou C, Sante T, Dheedene A, Heindryckx B, et al Whole genome amplification with SurePlex results in better copy number alteration detection using sequencing data compared to the MALBAC method. Sci Rep. 2015;5: 11711 10.1038/srep11711 26122179 PMC4485032

[21] Yan L, Huang L, Xu L, Huang J, Ma F, Zhu X, et al Live births after simultaneous avoidance of monogenic diseases and chromosome abnormality by next-generation sequencing with linkage analyses. Proc Natl Acad Sci U S A. 2015;112: 15964–9. 10.1073/pnas.1523297113 26712022 PMC4702982

[22] Blanco L, Bernad A, Lázaro JM, Martín G, Garmendia C, Salas M. Highly efficient DNA synthesis by the phage phi 29 DNA polymerase. Symmetrical mode of DNA replication. J Biol Chem. 1989;264: 8935–40. Available: http://www.ncbi.nlm.nih.gov/pubmed/2498321 2498321

[23] Borgström E, Paterlini M, Mold JE, Frisen J, Lundeberg J. Comparison of whole genome amplification techniques for human single cell exome sequencing. Li Y, editor. PLoS One. 2017;12: e0171566 10.1371/journal.pone.0171566 28207771 PMC5313163

[24] Huang L, Ma F, Chapman A, Lu S, Xie XS. Single-Cell Whole-Genome Amplification and Sequencing: Methodology and Applications. Annu Rev Genomics Hum Genet. 2015;16: 79–102. 10.1146/annurev-genom-090413-025352 26077818

[25] Kumar G, Garnova E, Reagin M, Vidali A. Improved multiple displacement amplification with phi29 DNA polymerase for genotyping of single human cells. Biotechniques. 2008;44: 879–90. 10.2144/000112755 18533898

[26] Rius M, Obradors A, Daina G, Cuzzi J, Marquès L, Calderón G, et al Reliability of short comparative genomic hybridization in fibroblasts and blastomeres for a comprehensive aneuploidy screening: first clinical application. Hum Reprod. 2010;25: 1824–35. 10.1093/humrep/deq118 20488804

[27] Daina G, Ramos L, Obradors A, Rius M, del Rey J, Martinez-Pasarell O, et al Double-factor preimplantation genetic diagnosis: Monogenic and cytogenetic diagnoses analyzing a single blastomere. Prenat Diagn. 2015;35 10.1002/pd.4691 26389801

[28] Handyside AH, Harton GL, Mariani B, Thornhill AR, Affara N, Shaw M-A, et al Karyomapping: a universal method for genome wide analysis of genetic disease based on mapping crossovers between parental haplotypes. J Med Genet. 2010;47: 651–658. 10.1136/jmg.2009.069971 19858130

[29] Fan J, Wang L, Wang H, Ma M, Wang S, Liu Z, et al The clinical utility of next-generation sequencing for identifying chromosome disease syndromes in human embryos. Reprod Biomed Online. 2015;31: 62–70. 10.1016/j.rbmo.2015.03.010 25985995

[30] Tortoriello D V., Dayal M, Beyhan Z, Yakut T, Keskintepe L. Reanalysis of human blastocysts with different molecular genetic screening platforms reveals significant discordance in ploidy status. J Assist Reprod Genet. 2016; 10.1007/s10815-016-0766-5 27423662 PMC5125143

[31] Huang J, Yan L, Lu S, Zhao N, Xie XS, Qiao J. Validation of a next-generation sequencing–based protocol for 24-chromosome aneuploidy screening of blastocysts. Fertil Steril. 2016;105: 1532–1536. 10.1016/j.fertnstert.2016.01.040 26902859

[32] Fiorentino F, Biricik A, Bono S, Spizzichino L, Cotroneo E, Cottone G, et al Development and validation of a next-generation sequencing–based protocol for 24-chromosome aneuploidy screening of embryos. Fertil Steril. 2014;101: 1375–1382.e2. 10.1016/j.fertnstert.2014.01.051 24613537

[33] Ramos L, del Rey J, Daina G, Martinez-Passarell O, Rius M, Tuñón D, et al Does the S phase have an impact on the accuracy of comparative genomic hybridization profiles in single fibroblasts and human blastomeres? Fertil Steril. 2014;101: 488–95. 10.1016/j.fertnstert.2013.10.031 24314925

[34] Zong C, Lu S, Chapman AR, Xie XS. Genome-Wide Detection of Single-Nucleotide and Copy-Number Variations of a Single Human Cell. Science (80-). 2012;338: 1622–1626. 10.1126/science.1229164 23258894 PMC3600412

[35] de Bourcy CFA, De Vlaminck I, Kanbar JN, Wang J, Gawad C, Quake SR. A quantitative comparison of single-cell whole genome amplification methods. Wang K, editor. PLoS One. 2014;9: e105585 10.1371/journal.pone.0105585 25136831 PMC4138190

[36] Strien J, Sanft J, Mall G. Enhancement of PCR Amplification of Moderate GC-Containing and Highly GC-Rich DNA Sequences. Mol Biotechnol. 2013;54: 1048–1054. 10.1007/s12033-013-9660-x 23568183

[37] Biesecker LG, Shianna K V, Mullikin JC. Exome sequencing: the expert view. Genome Biol. 2011;12: 128 10.1186/gb-2011-12-9-128 21920051 PMC3308041

[38] Natesan SA, Bladon AJ, Coskun S, Qubbaj W, Prates R, Munne S, et al Genome-wide karyomapping accurately identifies the inheritance of single-gene defects in human preimplantation embryos in vitro. Genet Med. 2014;16: 838–845. 10.1038/gim.2014.45 24810687 PMC4225458

[39] Codina-Pascual M, Campillo M, Kraus J, Speicher MR, Egozcue J, Navarro J, et al Crossover frequency and synaptonemal complex length: their variability and effects on human male meiosis. Mol Hum Reprod. 2006;12: 123–133. 10.1093/molehr/gal007 16449239

[40] Capalbo A, Treff NR, Cimadomo D, Tao X, Upham K, Ubaldi FM, et al Comparison of array comparative genomic hybridization and quantitative real-time PCR-based aneuploidy screening of blastocyst biopsies. Eur J Hum Genet. 2015;23: 901–906. 10.1038/ejhg.2014.222 25351780 PMC4463508

[41] Scott RT, Upham KM, Forman EJ, Zhao T, Treff NR. Cleavage-stage biopsy significantly impairs human embryonic implantation potential while blastocyst biopsy does not: a randomized and paired clinical trial. Fertil Steril. 2013;100: 624–30. 10.1016/j.fertnstert.2013.04.039 23773313

