# Supplementary material for: Novel Double Factor PGT strategy analyzing blastocyst stage embryos in a single NGS procedure
Source: PLoS One. 2018 Oct 17;13(10):e0205692. doi: 10.1371/journal.pone.0205692 (PMC6192598; doi:10.1371/journal.pone.0205692)
Supplement: S1 Table — (DOCX) [file pone.0205692.s001.docx]

| Select data type | BAM mCGH (matched) |
| --- | --- |
|  |  |
| **BAF Parameters** | |
| Reject reads wittl read depth less than | 200 |
| Reject reads with MAPQ less than | 200 |
| Reject bases with base quality less than | 200 |
| SNP file for BAf | dbSNPs.bed.bin |
| Remove homozygous reference probes | √ |
|  |  |
| **Coversion** | |
| reference reads per CN point | 10000 |
|  |  |
| **Systematic correction** | |
| Type | Linear correction |
| File | ngs_correction.txt |
|  |  |
| **Recenter Probes** | |
| Type | Median |
|  |  |
| **Analysis** | |
| Type: | FASST2 Segmentation |
| Significance Threshold | 1.0E-7 |
| Max Contiguous Probe Spacing (Kbp) | 1000 |
| Min number of probes per segment | 10 |
| High Gain | 0.6 |
| Gain | 0.35 |
| Loss | -0.35 |
| Big Loss | -1.0 |
| Male Sex Chromosomes Big Loss | -1.0 |
| 3:1 Sex chromosome gain | 1.2 |
| 4:1 Sex chromosome gain | 1.7 |
|  |  |
| **Robust Variance Sample QC Calculation** | |
| Percent outliers to remove | 5.0 |

S1 Table. Nexus software settings for CNV analysis
