# Supplementary figures and images for: Novel Double Factor PGT strategy analyzing blastocyst stage embryos in a single NGS procedure
Source: PLoS One. 2018 Oct 17;13(10):e0205692. doi: 10.1371/journal.pone.0205692 (PMC6192598; doi:10.1371/journal.pone.0205692)

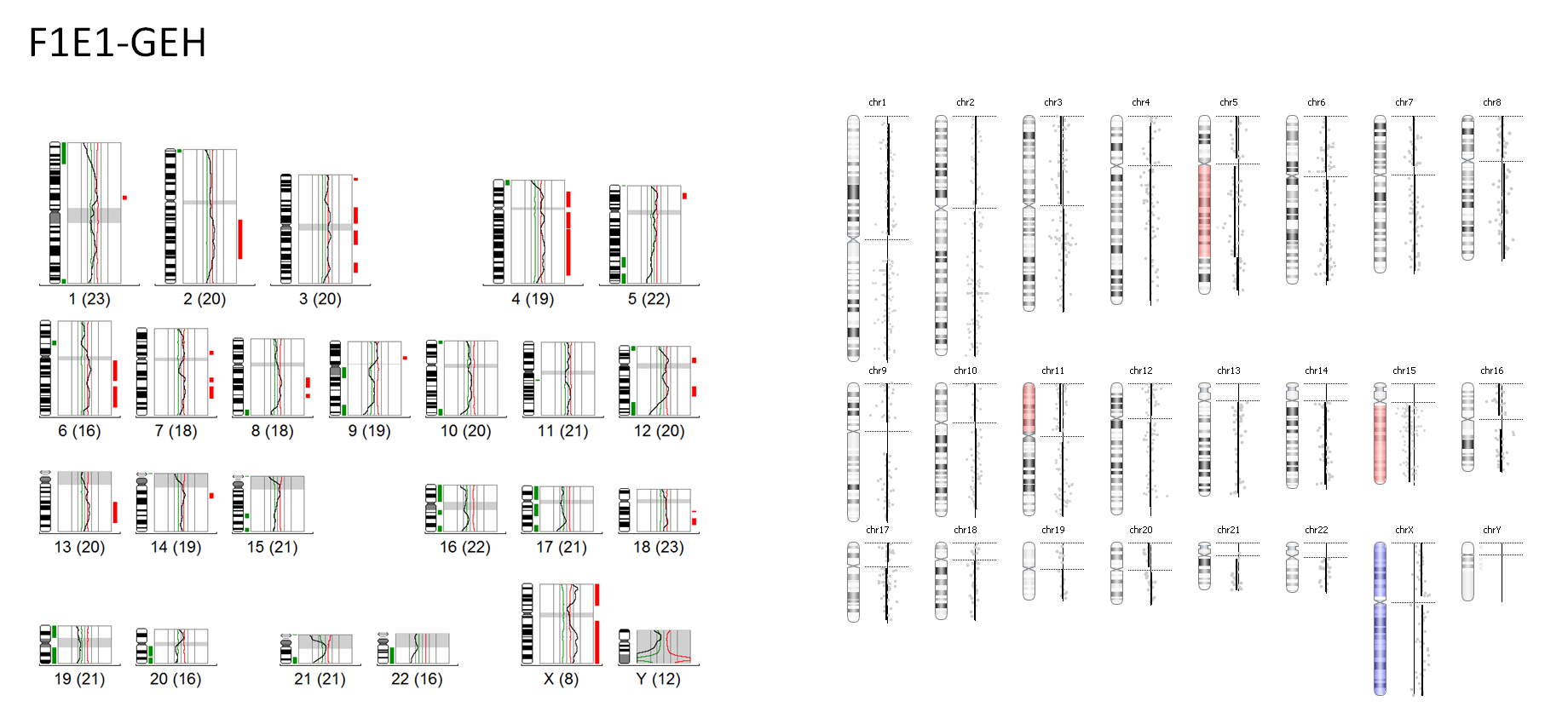

Supplement: S1 Fig — On the left, mCGH results, reference used 46, XY. On the right, NGS-CNV summary plot, reference used 47,XY+15. Note that the loss of chromosome 15 observed is due to the reference used. (TIF) [file pone.0205692.s002.tif]

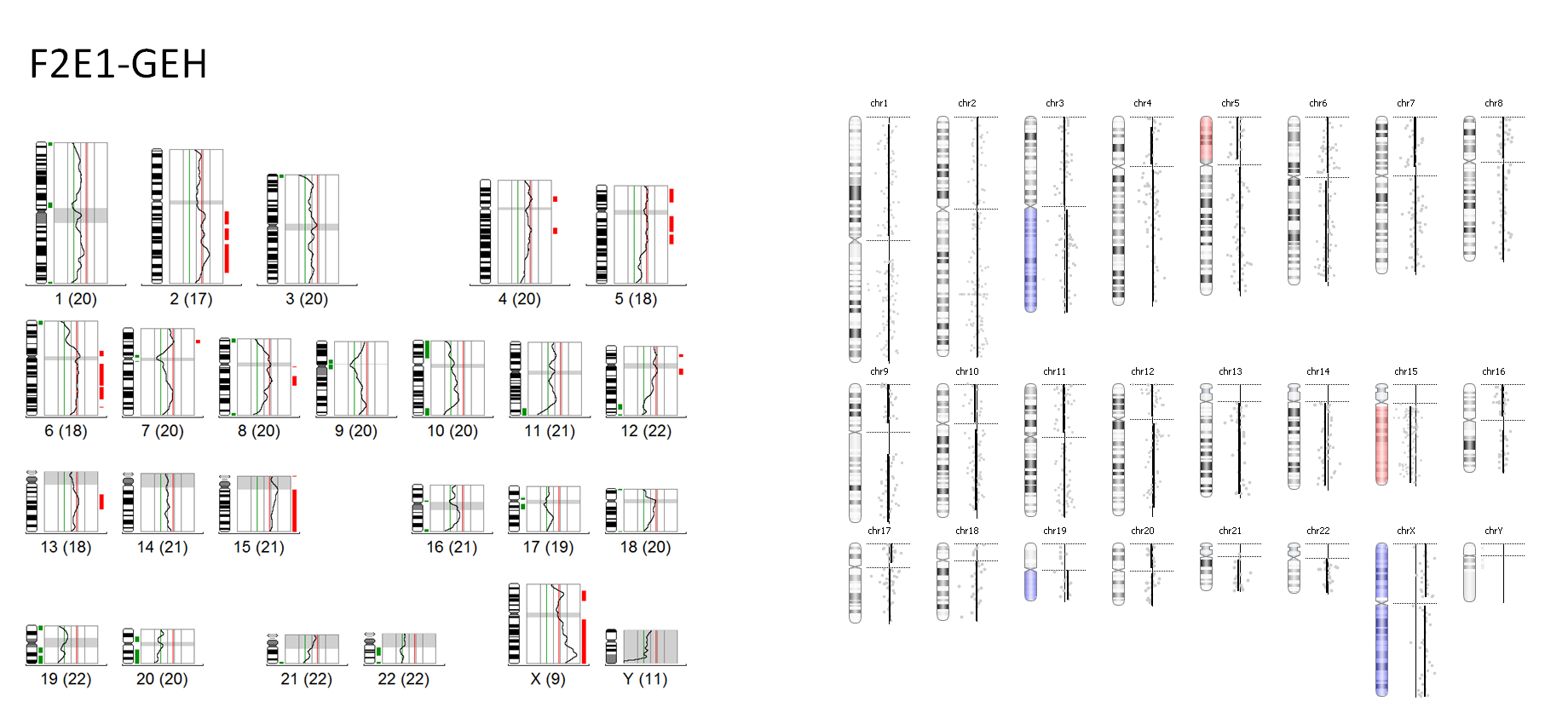

Supplement: S2 Fig — On the left, mCGH results, reference used 46, XY. On the right, NGS-CNV summary plot, reference used 47,XY+15. Note that the loss of chromosome 15 observed is due to the reference used. (TIF) [file pone.0205692.s003.tif]

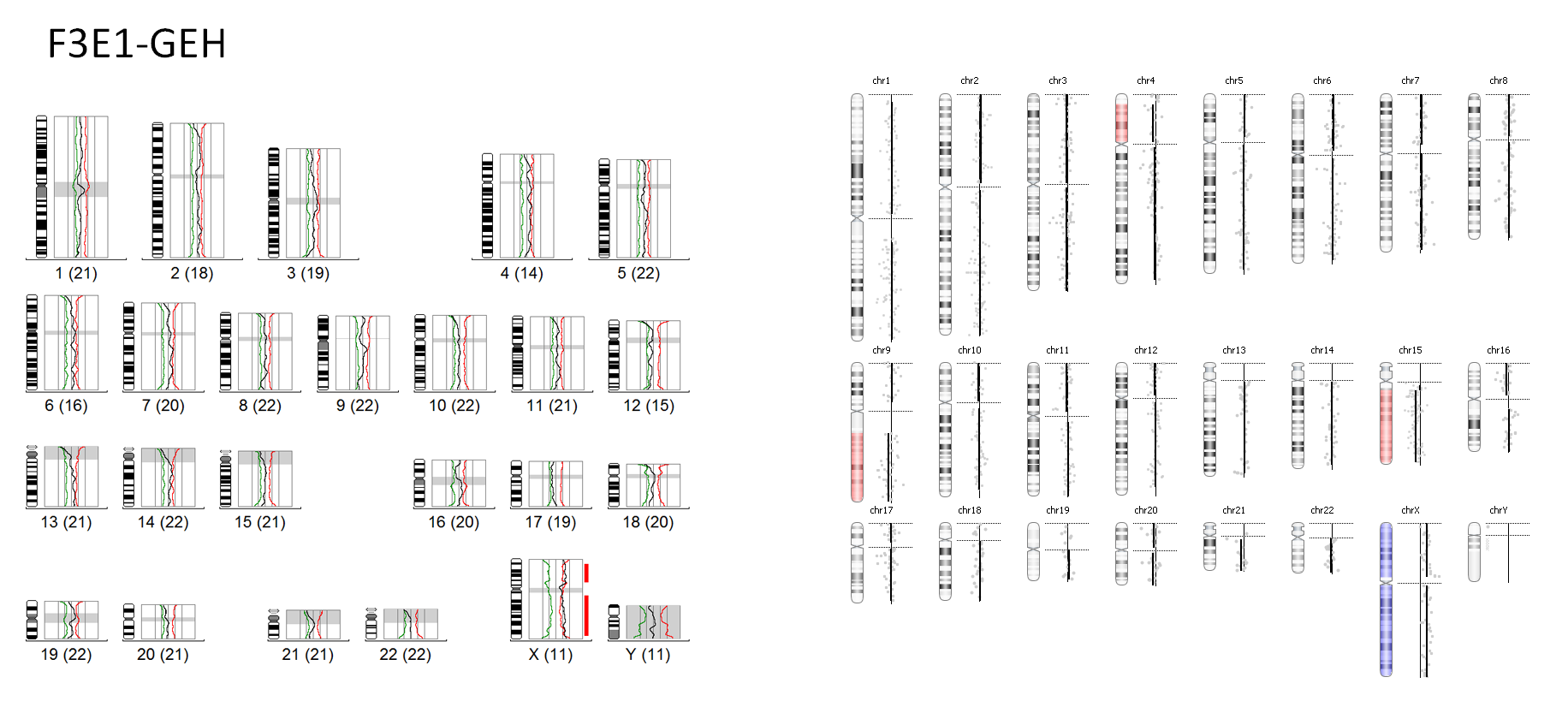

Supplement: S3 Fig — On the left, mCGH results, reference used 46, XY. On the right, NGS-CNV summary plot, reference used 47,XY+15. Note that the loss of chromosome 15 observed is due to the reference used. (TIF) [file pone.0205692.s004.tif]

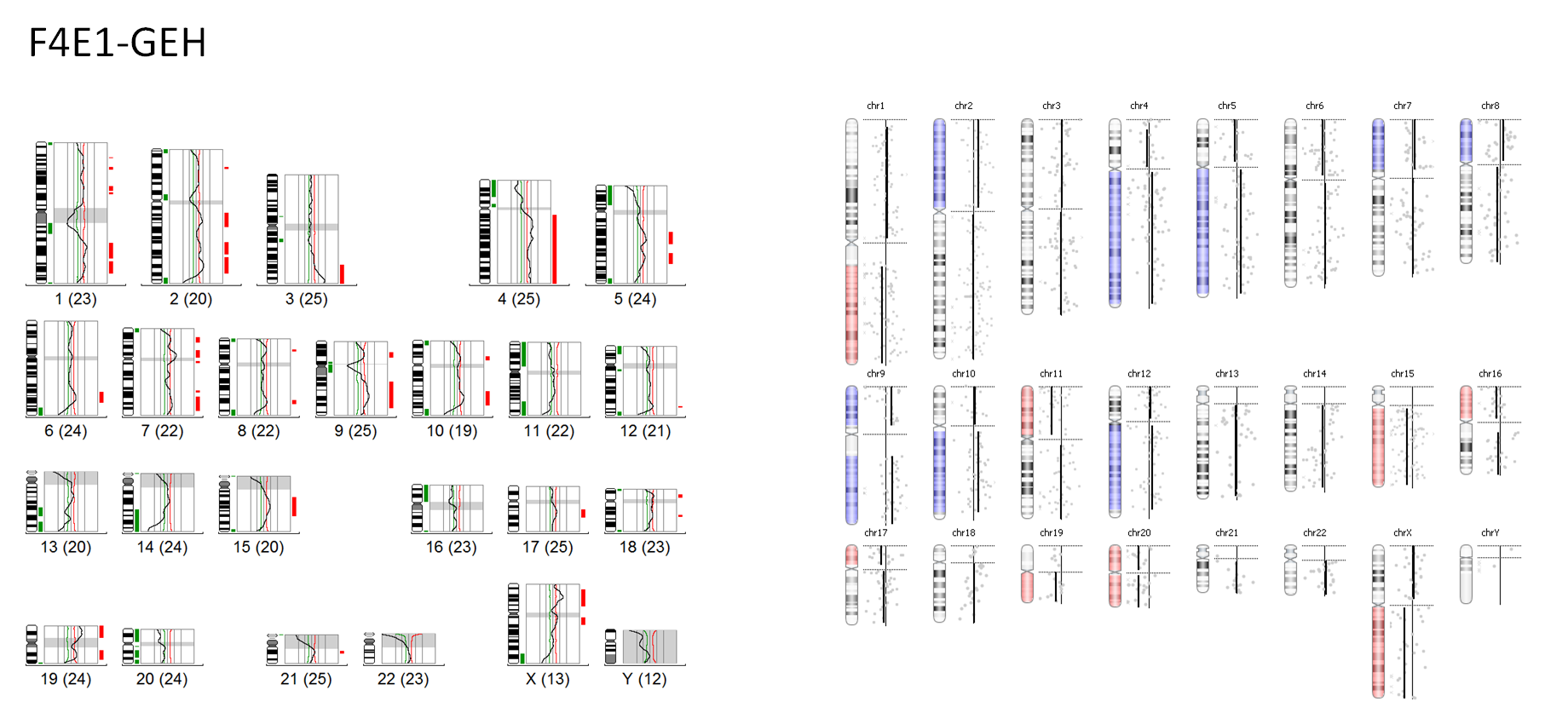

Supplement: S4 Fig — On the left, mCGH results, reference used 46, XY. On the right, NGS-CNV summary plot, reference used 47,XY+15. Note that the loss of chromosome 15 observed is due to the reference used. (TIF) [file pone.0205692.s005.tif]

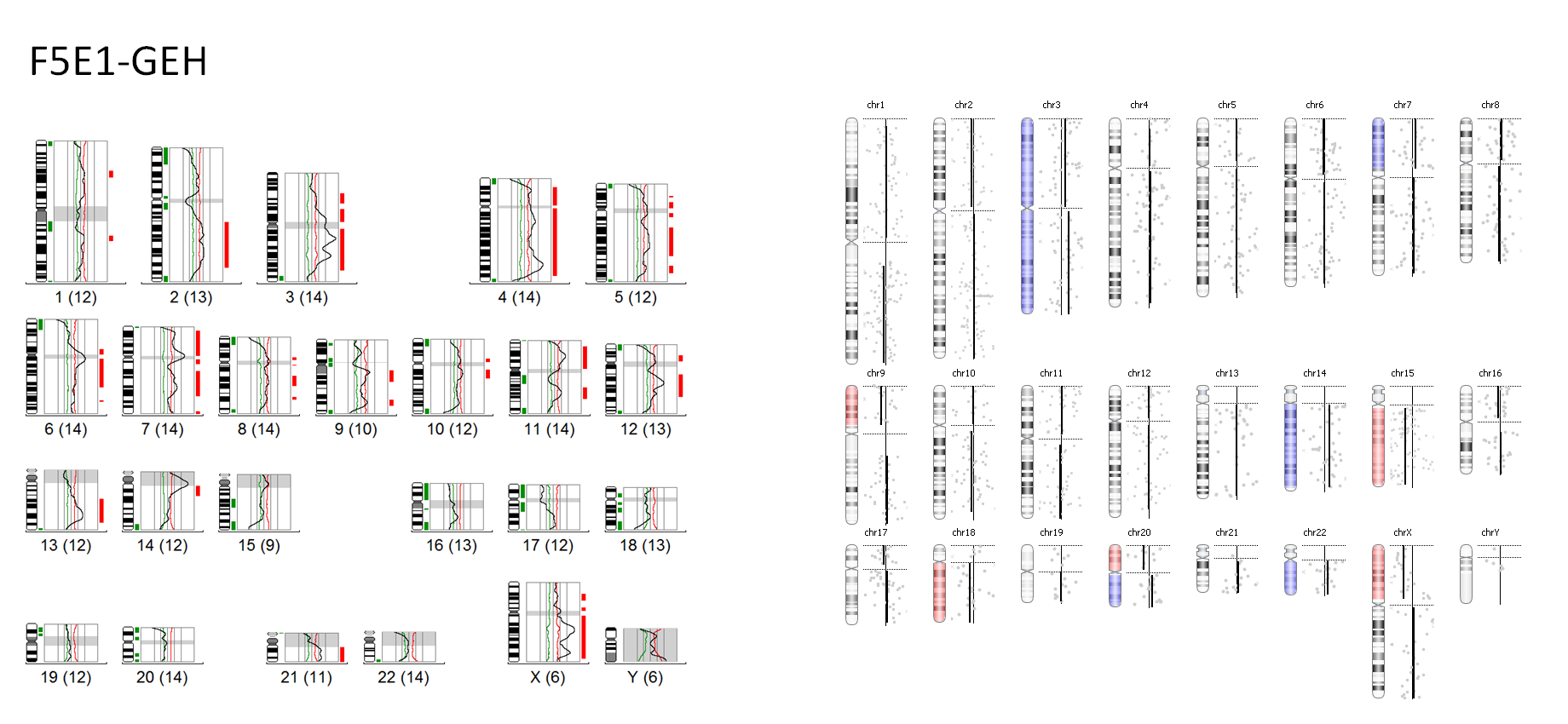

Supplement: S5 Fig — On the left, mCGH results, reference used 46, XY. On the right, NGS-CNV summary plot, reference used 47,XY+15. Note that the loss of chromosome 15 observed is due to the reference used. (TIF) [file pone.0205692.s006.tif]

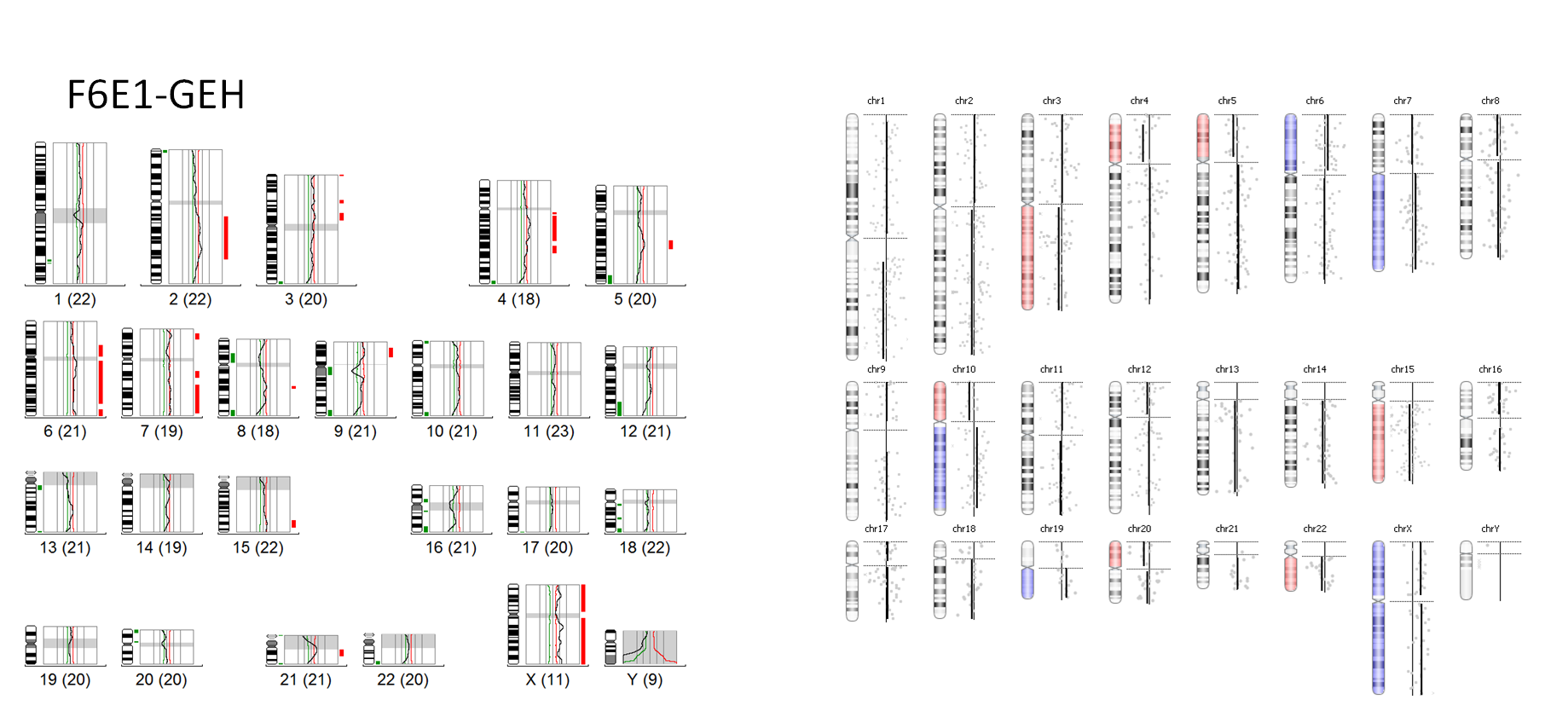

Supplement: S6 Fig — On the left, mCGH results, reference used 46, XY. On the right, NGS-CNV summary plot, reference used 47,XY+15. Note that the loss of chromosome 15 observed is due to the reference used. (TIF) [file pone.0205692.s007.tif]

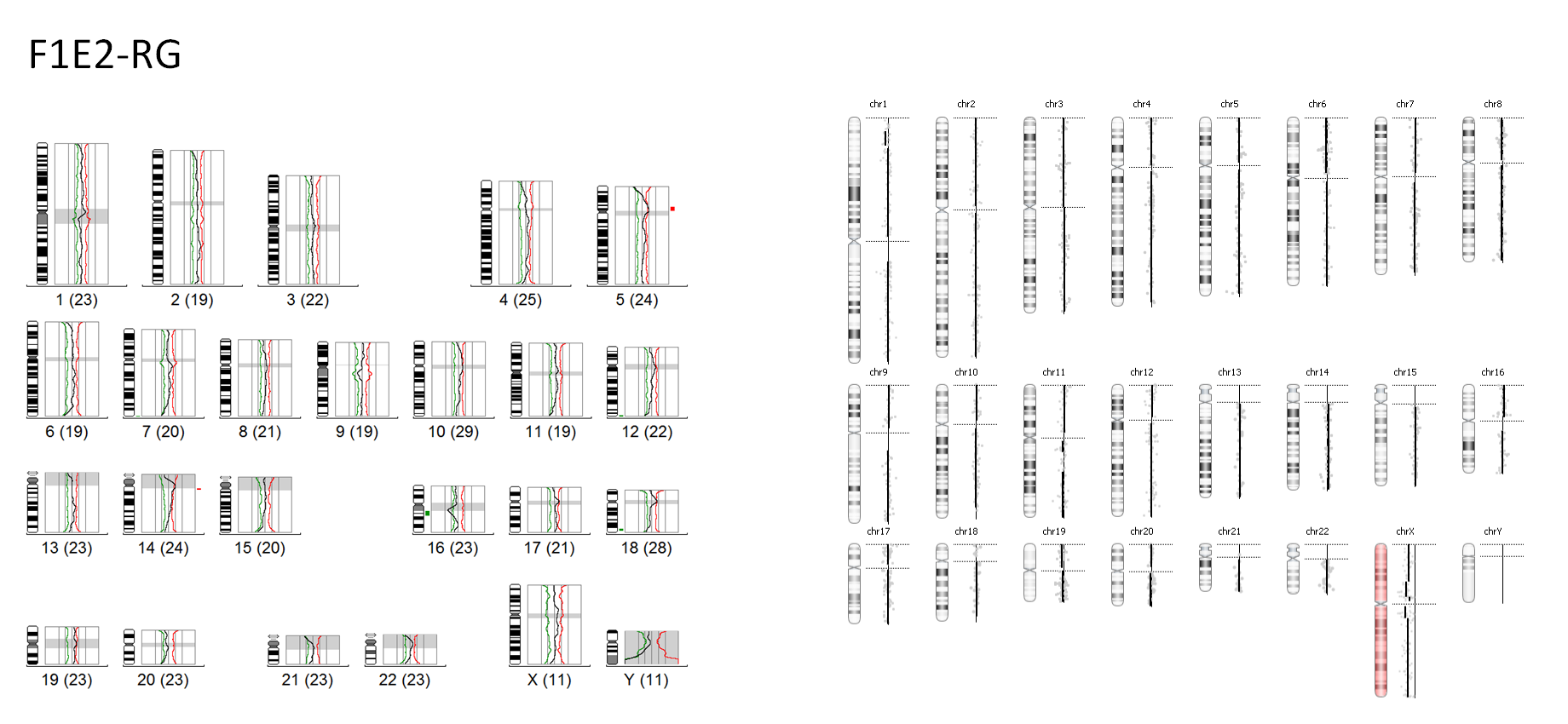

Supplement: S7 Fig — On the left, mCGH results, reference used 46, XY. On the right, NGS-CNV summary plot, reference used 46,XX. (TIF) [file pone.0205692.s008.tif]

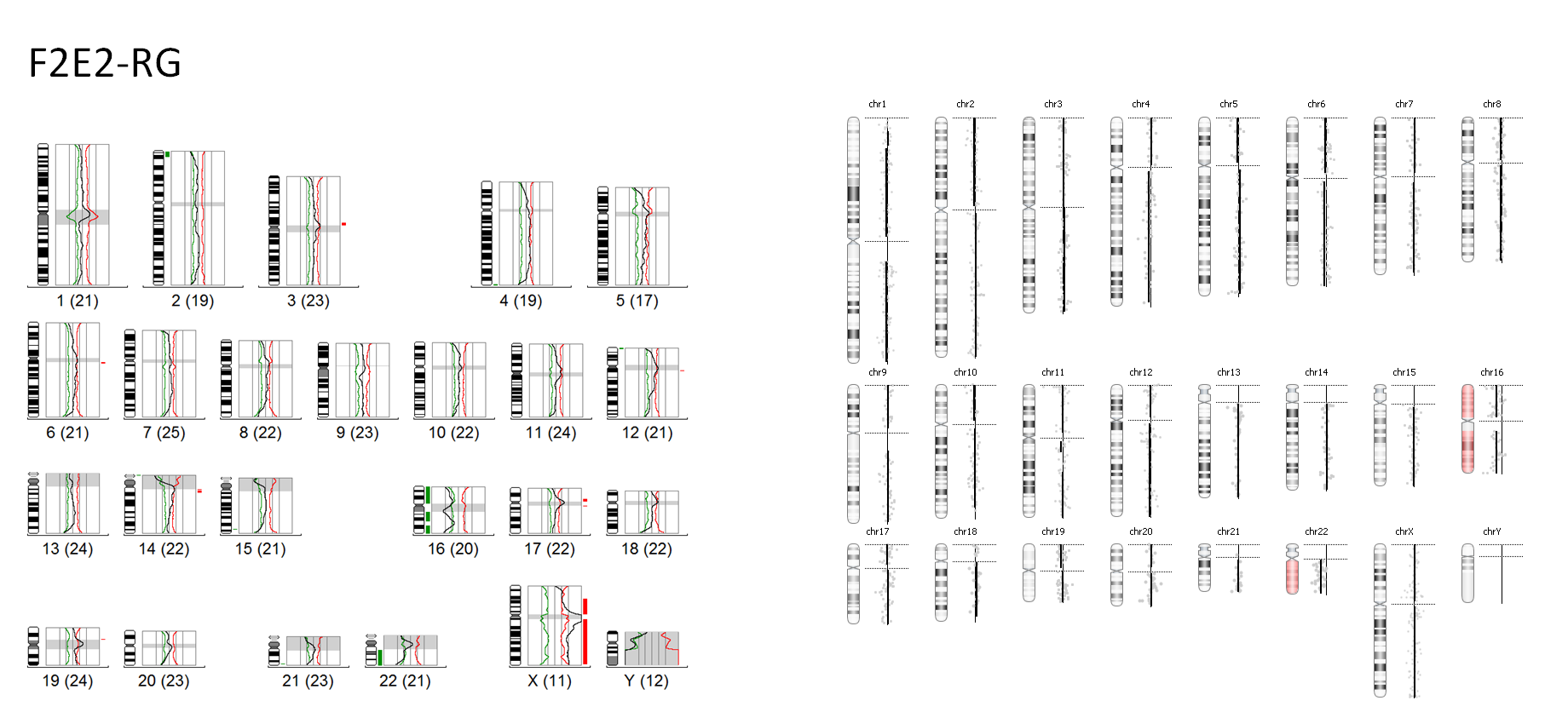

Supplement: S8 Fig — On the left, mCGH results, reference used 46, XY. On the right, NGS-CNV summary plot, reference used 46,XX. (TIF) [file pone.0205692.s009.tif]

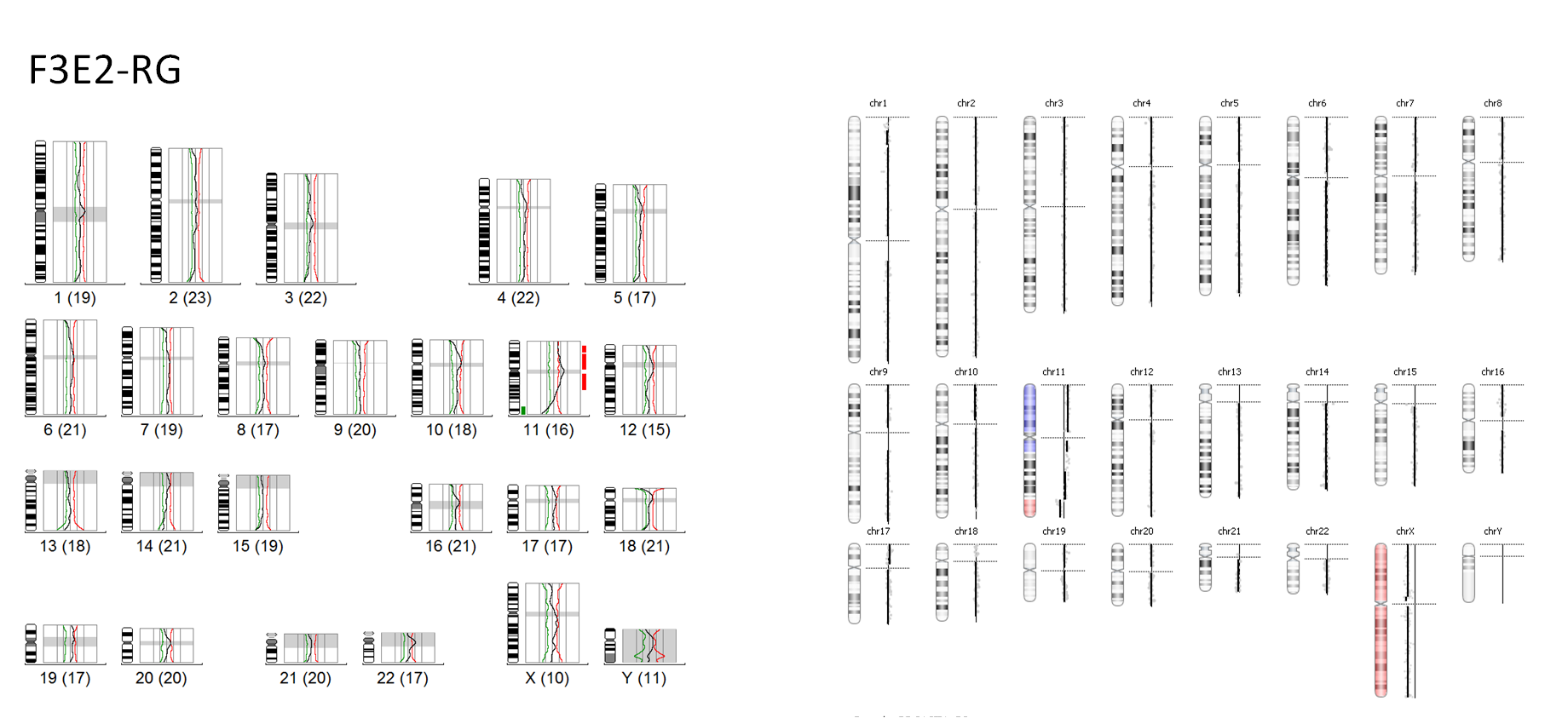

Supplement: S9 Fig — On the left, mCGH results, reference used 46, XY. On the right, NGS-CNV summary plot, reference used 46,XX. (TIF) [file pone.0205692.s010.tif]

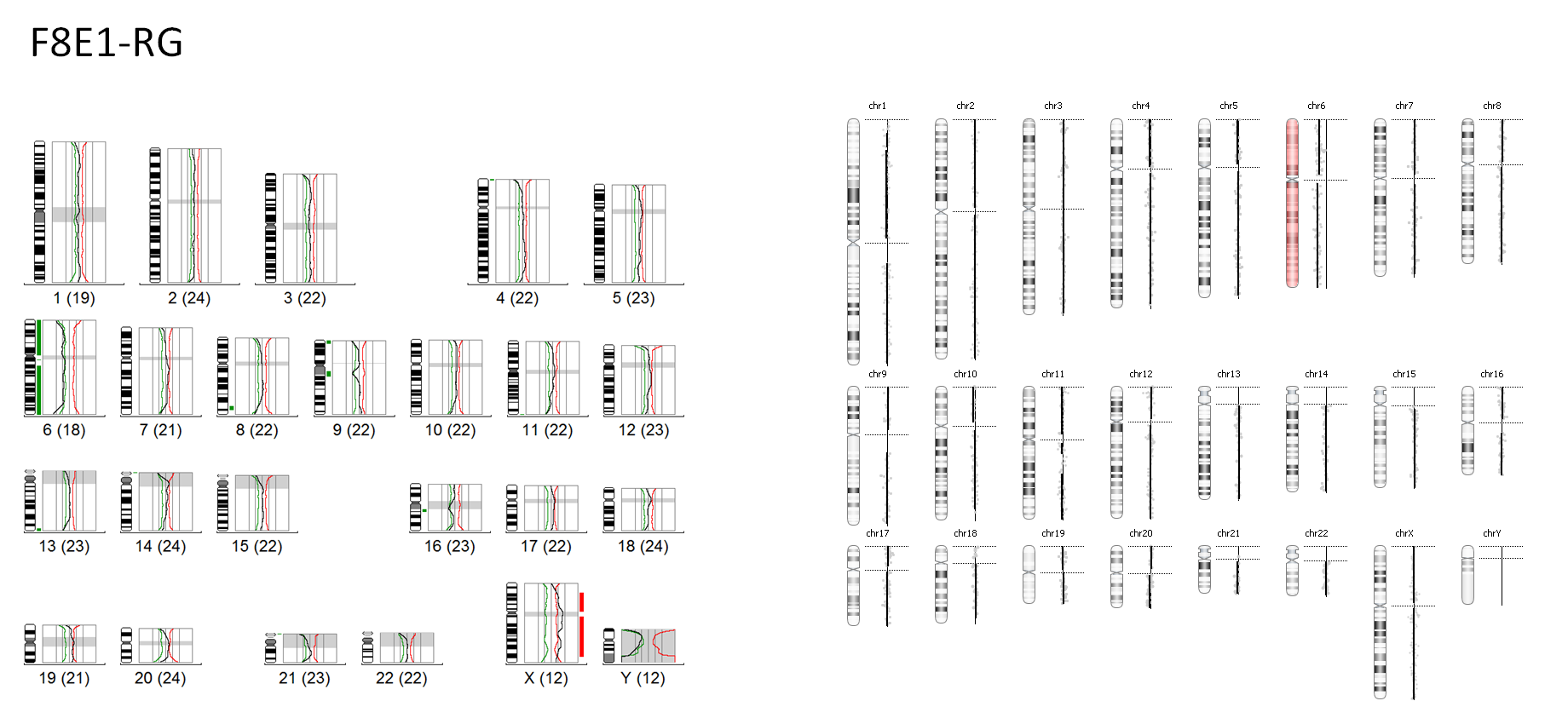

Supplement: S10 Fig — On the left, mCGH results, reference used 46, XY. On the right, NGS-CNV summary plot, reference used 46,XX. (TIF) [file pone.0205692.s011.tif]

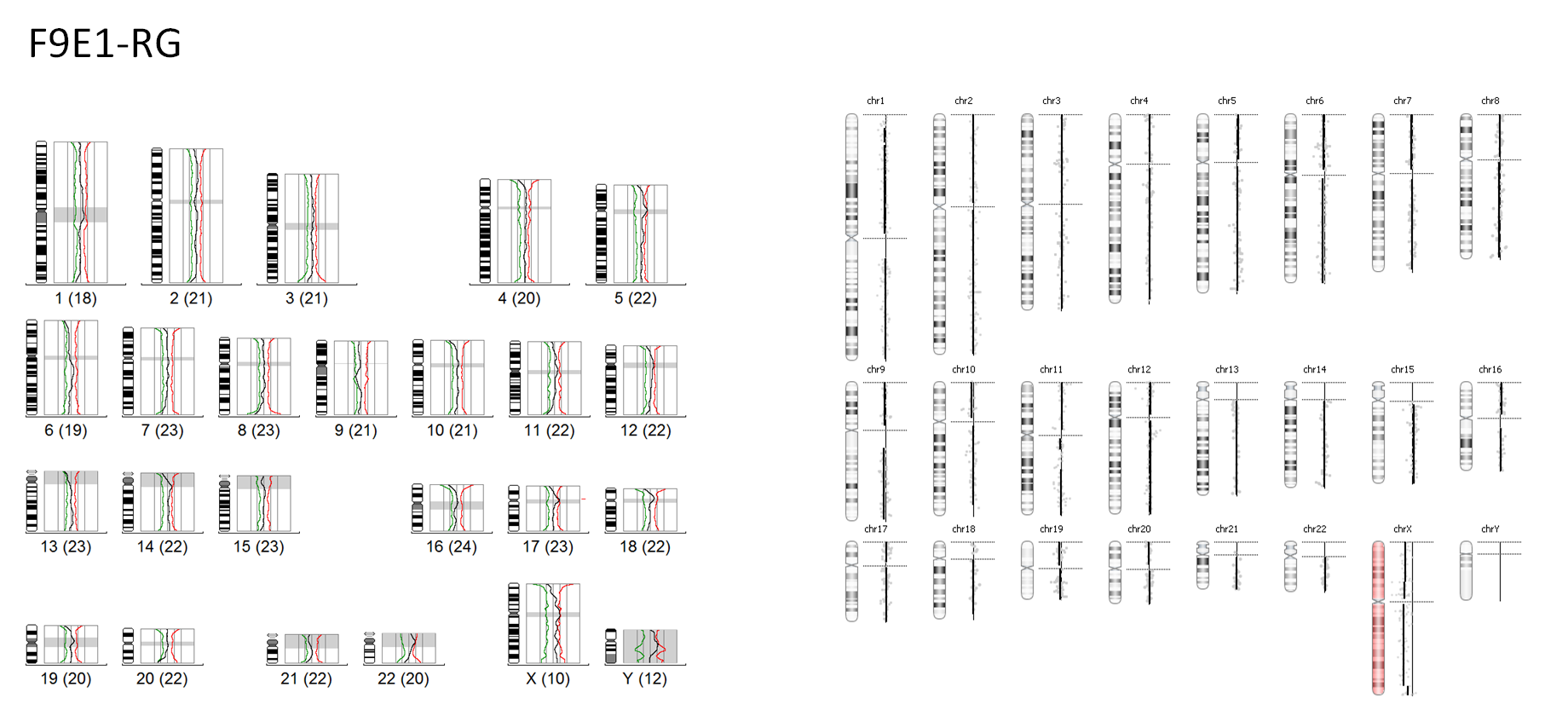

Supplement: S11 Fig — On the left, mCGH results, reference used 46, XY. On the right, NGS-CNV summary plot, reference used 46,XX. (TIF) [file pone.0205692.s012.tif]

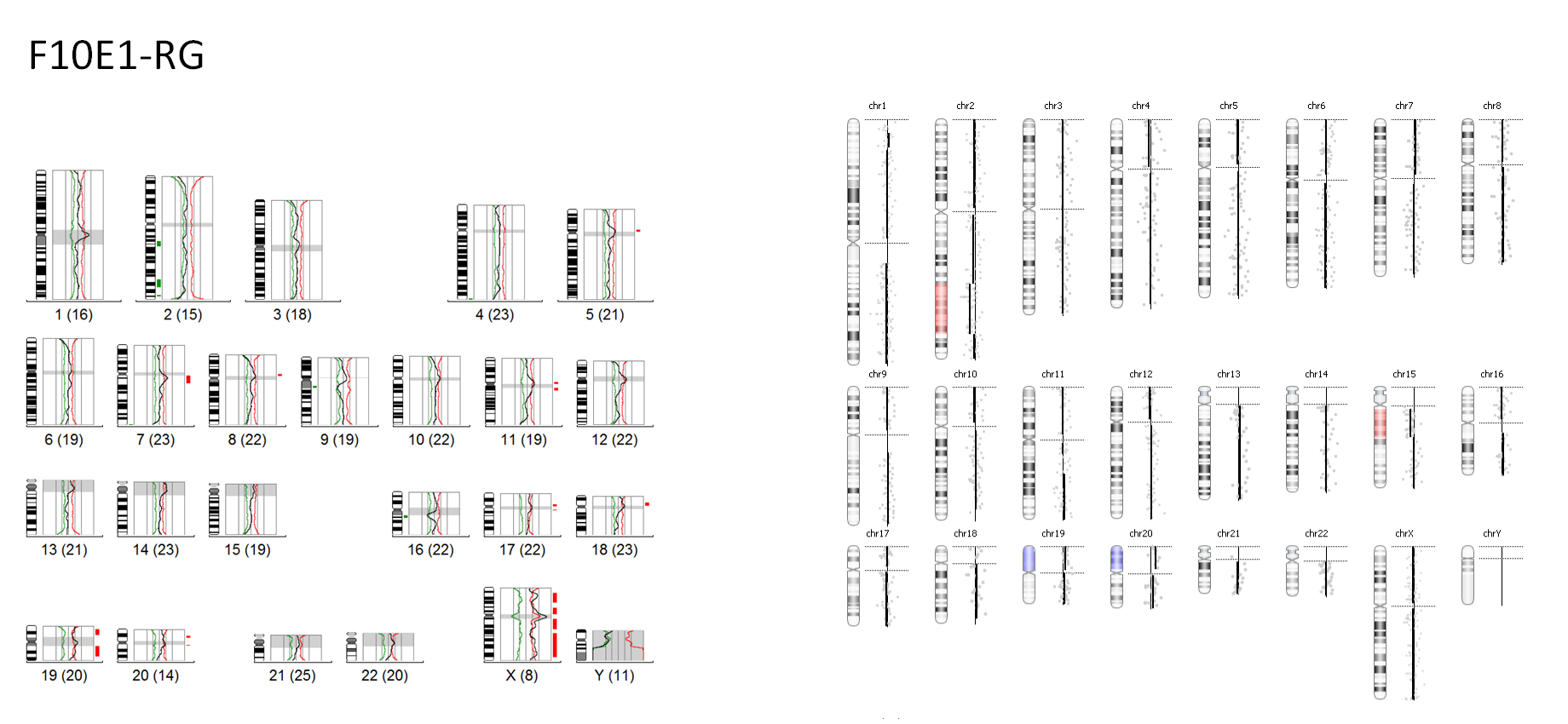

Supplement: S12 Fig — On the left, mCGH results, reference used 46, XY. On the right, NGS-CNV summary plot, reference used 46,XX. (TIF) [file pone.0205692.s013.tif]
